# Unravelling pain in Göttingen Minipigs undergoing experimentally induced closed-chest myocardial infarction: a prospective cohort study

**DOI:** 10.1038/s41598-025-20920-y

**Published:** 2025-10-22

**Authors:** Mariafrancesca Petrucci, Anne-Christine Uldry, Chiara Parodi, Luisana Gisela Garcia Casalta, Alain Despont, Noé Corpataux, Fabien Praz, Robert Rieben, Daniela Casoni

**Affiliations:** 1 Experimental Surgery Facility (ESF), Experimental Animal Center (EAC), Faculty of Medicine, University of Bern, Bern, Switzerland; 2 Graduate School for Cellular and BioMedical Science, University of Bern, Bern, Switzerland; 3 Department for BioMedical Research, Faculty of Medicine, University of Bern, Bern, Switzerland; 4 Department of Clinical Sciences, Faculty of Veterinary Medicine, Université de Montréal, Saint-Hyacinthe, QC Canada; 5 Proteomics and Mass Spectrometry Core Facility, Department for BioMedical Research, University of Bern, Bern, Switzerland; 6 Institute of Physiology, Faculty of Medicine, University of Bern, Bern, Switzerland; 7 Department of Cardiology, Inselspital, University of Bern, Bern, Switzerland

**Keywords:** Pain, Cardiovascular disease, Biomedical research, Swine, Quantitative sensory testing, Behaviours, Cardiology, Medical research, Physiology

## Abstract

The pain associated with experimental myocardial infarction in pigs has never been investigated. We aimed at assessing pain and its correlation with myocardial damage. Twenty-four Göttingen minipigs undergoing closed chest myocardial infarction followed by coronary reperfusion under general balanced anaesthesia were included in the trial. Pain was assessed through mechanical and thermal thresholds, sensitivity to Von Frey filaments and behavioural indicators before (Pre MI), the day after (Post MI) and at the study endpoint (Post MI-endpoint). Over time differences in mechanical thresholds (MT) and thermal thresholds (TT) were assessed using one-sample t-test and their correlations with troponin I/cytokines using logistic regression. In four minipigs at Post MI acute pain requiring analgesia was identified. Pain thresholds decreased significantly at Post MI (MT: 51 [35.6; 74] TT: 44.8 [42.7; 48.7]) and Post MI-endpoint (MT: 47.5 [35; 64.3]; TT: 44.3 [43.1; 48.6]) compared to Pre MI (MT: 72 [53.4; 84], TT: 46.3 [43.8; 53.8]). The response to von Frey filaments remained sporadic. Troponin I highly increased at Post MI, but no correlations with pain thresholds were found. Following balanced anaesthesia, acute pain had low incidence and mild to moderate intensity. Somatic hyperalgesia remained until the study endpoint, but its relevance remains to be unravelled.

**Supplementary Information:**

The online version contains supplementary material available at 10.1038/s41598-025-20920-y.

## Introduction

Acute myocardial infarction (MI) is a life-threatening condition characterised by myocardial cell death in response to myocardial ischaemia due to coronary occlusion or significant stenosis which causes more than 6 million deaths worldwide annually^1,2^. If not causing sudden death, MI can lead to heart failure^3^, which in turn is associated with significant decline in quality of life (4) and tremendous impact on healthcare system . The current best therapeutic choice for patients suffering of myocardial infarction is the percutaneous coronary intervention (PCI)^5^. Despite allowing revascularisation of the coronary arteries, this technique does not prevent the development of fibrosis and chronic heart failure^6^. Therefore, therapeutic solutions to reduce the long-term consequences of MI are still highly required and necessitate preclinical studies on animal models^7^.

Pain is a typical and frequent complaint in patients suffering from acute MI. Pain localisation, duration and intensity may differ among individuals and evidences indicate an important role played by gender and patients’ stress and personality^8–11^. The most common presentation is the angina pectoris, described as a pressure or burning sensation perceived at the level of the chest. Typically, pain also refers to shoulders, forearms, neck, jaw and the epigastric region^12^. Myocardial infarction can also occur in absence of pain; in this case, it is defined as silent myocardial infarction^13^. After percutaneous coronary intervention (PCI) chest pain has been observed in patients within 24 h after the intervention^14,15^ and persistent pain one year after the occurrence of the disease has also been reported^16^.

Due to their anatomical and physiological similarity to humans, pigs and minipigs are frequently used as translational models for investigating therapeutic strategies against MI and its consequences^17–19^ . For the same reason, they have been proposed as valuable alternative to rodents in pain research^20^ . Nevertheless, investigations on pain experienced by pigs and minipigs undergoing experimentally induced MI are lacking^21^, undoubtedly raising an important ethical issue. Furthermore, since animals are not aware of the disease’s severity, data might also help to understand pain mechanisms in humans, cleansed by the influence of catastrophising.

Quantitative sensory testing (QST) assess the somatosensory function of the pain pathway^22^ and help objectivizing alterations in pain perception in response to afferent stimuli (e.g., hyperalgesia, allodynia), in humans^23^ and veterinary species^24–29^. While only few studies have investigated QST in humans suffering from MI^8,30–32^, none have yet examined its use in animals enrolled in cardiovascular research.

Behavioural evaluation as a way of assessing pain is gaining importance in veterinary medicine^33–35^. The UNESP-Botucatu scale has been recently validated for husbandry procedures in piglets^36,37^ but no scale has been proposed nor validated in adult minipigs undergoing experimental procedures.

Cytokines such as Tumour Necrosis Factor Alpha (TNFα), Interleukin 6 and 1 Beta (IL6 and IL1β, respectively) have been proposed as potential biomarkers for acute pain in calves and humans^38–43^, but their role is still not fully elucidated^42,43^. The same cytokines increase in patients following MI^44^, due to occurrence of ischaemia/reperfusion injury and consequent inflammatory reaction and oxidative stress^45,46^. Whether they could represent a biomarker of pain after myocardial infarction in minipigs is unknown.

The overarching aim of this study was to unravel pain in Göttingen Minipigs undergoing experimentally induced closed-chest MI. Specifically, we aimed at: 1) assessing the variations of QST thresholds (mechanical, thermal and Von Frey) and behaviours associated with pain in the immediate post operative period (Post MI) and before the study endpoint (42 ± 3 days, Post MI-endpoint) compared to values recorded before the procedure (Pre MI); 2) correlating QST variation between days with behaviours associated with pain, cardiac troponin I (reflecting myocardial damage) and cytokines (reflecting inflammation).

We hypothesized that 1) QST thresholds would decrease following MI induction and will correlate with increased frequency of behaviours associated with pain; 2) no correlations would be found between QST and the severity of myocardial damage/inflammation.

## Results

Twenty-four animals (age: 15.5 ± 2 months, body weight: 30.4 ± 4.4), 11 females (age: 14 ± 3 months, body weight: 29.2 ± 4.5 kg) and 13 males (age: 16 ± 1 months, body weight: 31.5 ± 4 kg) completed data collection and were included in the statistical analysis.

Three minipigs (2 males and 1 female) were excluded as they did not survive the coronary ischaemia.

In four animals, it was not possible to withdraw blood from the port-catheter at Post MI-endpoint (day 35 ± 2), but arterial blood was withdrawn from a catheter placed in the coccygeal artery at the study endpoint (day 42 ± 3). In an animal the port-catheter was not implanted, and only arterial blood was collected (at Pre MI: in general anaesthesia, after arterial catheterisation before the procedure; at Post MI: from the arterial catheter positioned the day before; at Post MI-endpoint: after induction of terminal general anaesthesia, day 42 ± 3).

Only descriptive analysis was performed for the Von Frey data, due to the paucity of obtained responses. Raw results for each day and minipig are reported in the supplementary materials (supplementary file S1).

### Mechanical and thermal thresholds

Median and interquartile range (IQR) [25^th^; 75^th^] of mechanical thresholds (MT) and thermal thresholds (TT) for each day were:

- Whole sample:

Pre MI → MT: 72 [53.4; 84]; TT: 46.3 [43.8; 53.8]

Post MI → MT: 51 [35.6; 74]; TT: 44.8 [42.7; 48.7]

Post MI-endpoint → MT: 47.5 [35; 64.3]; TT: 44.3 [43.1; 48.6]

- Females:

Pre MI → MT: 71.3 [44.8; 91.6]; TT: 45.8 [43.3; 50.5]

Post MI → MT: 43.8 [30; 66.6]; TT: 43.8 [42.3; 46.6]

Post MI-endpoint → MT: 43.3 [32.4; 73.1]; TT: 44.7 [42.9; 49.8].

- Males:

Pre MI → MT: 73 [54.9; 81.3]; TT: 47.2 [44.9; 56]

Post MI → MT: 59 [44.3; 79]; TT: 46 [43.5; 54.8]

Post MI-endpoint → **MT**: 52.8 [39.3; 63.5]; **TT**: 44.3 [43.1; 48.3].

**Fig. 1:**
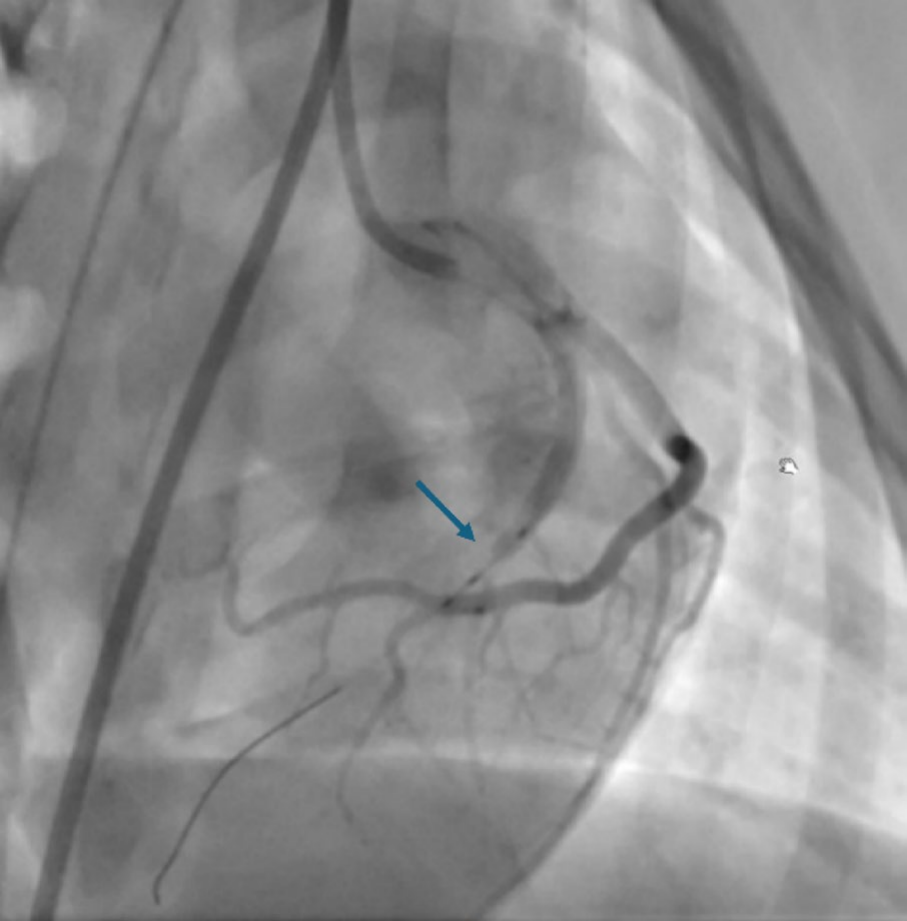
Density plot showing mechanical thresholds modifications for each day (Pre MI, Post MI and Post MI-endpoint), in 24 minipigs, for all sites (total = 144 measurements). X-axis: Newton (0–101); y-axis: days.

**Fig. 2:**
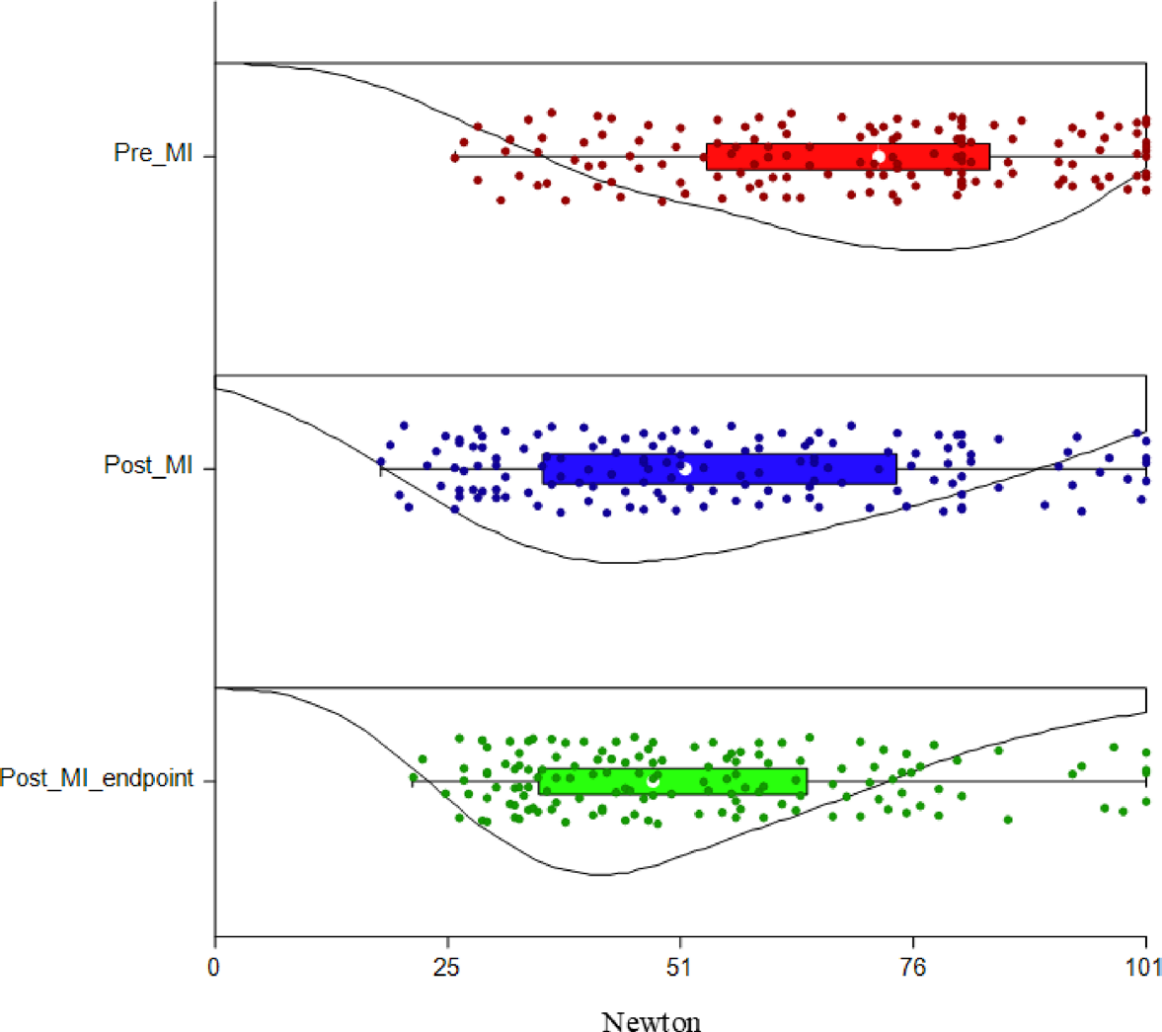
Density plot showing thermal thresholds modifications for each day (Pre MI, Post MI and Post MI-endpoint), in 24 minipigs, for all sites (total = 144 measurements). X-axis: degrees Celsius (20–56); y-axis: days.

Density plots of MT and TT recorded for each day, for the whole sample are reported in Figs. 1 and 2.

Box plots of MT and TT recorded for each minipig (with indication of sex) and day are reported in supplementary file S2 (Supplementary Figs S1-S7).

- Pre MI versus Post MI

A statistically significant decrease of MT and TT was found in the whole sample (p = 0.0014 and p = 0.028, respectively) when considering the median of paired differences. Mechanical thresholds showed a larger effect size compared to TT (d = − 0.74 and d = − 0.48, respectively). When sex was considered, females showed a statistically significant difference in MT (p = 0.0098, d = − 0.96) and TT (p = 0.046, d = − 0.69). On the contrary, males showed no differences in both MT (p = 0.068, d = − 0.56) and TT (p = 0.32, d = − 0.29). After correcting for multiple testing, a statistically significant decrease was found only for MT in the whole sample (p = 0.008), and in females (p = 0.02) as measured by the median of site-paired differences. Further details are reported in supplementary file S3.

- Pre MI versus Post MI- endpoint

A statistically significant decrease in MT and TT was found in the whole sample (p < 0.001 and p = 0.025, respectively). Also in this case, a larger effect size was present for MT compared to TT (d = 0.93 and d = 0.49, respectively). When sex was considered, females showed a statistically significant decrease in MT (p = 0.025, d = 0.79) but not in TT (p = 0.63, d = 0.15). On the contrary, males showed a statistically significant decrease in both MT (p = 0.0017, d = 1) and TT (p = 0.0030, d = 1). These results remain valid after correcting for multiple testing. Further details are reported in supplementary file S4.

- Post MI versus Post MI- endpoint

No differences in MT and TT were found in the whole sample (p = 0.43, d = 0.16 and p = 0.75, d = 0.06, respectively). When sex was considered, females did not show any differences both in MT (p = 0.6, d = − 0.12) and TT (p = 0.26, d = − 0.3). On the contrary, males showed a statistically significant decrease in TT (p = 0.0041, d = 0.98) but not in MT (p = 0.12, d = 0.45). These results remain valid after correcting for multiple testing. Further details are reported in supplementary file S5.

Mechanical and thermal thresholds recorded for each site and day are reported in Table 1.

**Table 1:** Mechanical and thermal thresholds recorded in all minipigs (n = 24) for each day (Pre MI, Post MI and Post MI-endpoint) and site.

Site	Pre MI	Post MI	Post MI- endpoint
Mechanical thresholds			
LF	81 [60.4; 98.3]	58.3 [38.4;90.8]	60.8 [37.6; 91]
RF	75 [58.1;95.5]	61.8 [37.9; 80.3]	52.8 [34.3; 75.3]
LC	61.3 [42.3; 81]	49.8 [41.6; 64.9]	46.3 [39.4; 56.9]
RC	70 [58.6; 79.1]	53 [41.5; 65.5]	56 [43.8; 66]
LN	67.3 [44.4; 81]	44 [26.6; 73.8]	39 [33.6; 60.9]
RN	73.8 [50.1;84.4]	46.5 [31.4; 73.6]	39.5 [34; 52.5]
Thermal thresholds			
LF	46.3 [45.1; 53.8]	45.6 [72.8; 56]	43.9 [43; 51.8]
RF	45.8 [43.3; 50.8]	46.4 [43.2; 54.7]	45.3 [44; 50.1]
LC	45.9 [44.5; 53.9]	43.2 [43; 46.2]	43.6 [43; 46.2]
RC	46.3 [41.5; 50.2]	44.6 [43.5; 49.9]	43.8 [42.5; 47.5]
LN	45.9 [42.9; 54.3]	44 [41.5; 48]	44.4 [42.8; 47.5]
RN	51.7 [45.8; 56]	45 [43.8; 48.7]	44.9 [43.4; 50.9]

Results are reported as median and interquartile range [25^th^; 75^th^]. LF: left forearm; RF: right forearm; LC: left chest; RC: right chest; LN: left neck; RN: right neck.

Results of the comparison between days for each site, both for the whole samples and for each sex, are reported in supplementary file S6.

- Pain scale assessment

Details on behavioural items used to assess pain are reported in Table 2 (Post MI) and Table 3 (Post MI-endpoint).

**Table 2:** Behavioural items recorded after the end of the procedure (Post MI). The score was obtained at multiple evaluations, starting from extubation until 12 h after sternal recumbency*.*

	Appearance**	Lying and restlessness **	Tremors/ Spasms	Vocalisation	Food interest	Agitation at human approaching *	Response to touch (neck, head, thorax and forelimbs) *
0	Normal appearance	Normal lying	No evidence of tremors/ spasms	No vocalisation or vocalisation related to food/interest in human/mate approach	Normal appetite	Curious, interactive, may vocalise	No response
1	Salivation	Lying guarding one part of the body/moves without external stimulation	Tremors/ spasms in one part of the body	Vocalisation without a purpose/ unmotivated excitation	Reduced appetite, eat special/ tasty food	Moves away when approached	Mild response: the animal looks uncomfortable and worried; retraction of the interested body part can be present
2	//	Move often/ poor wake times	Generalised tremors/ spasms	//	Reduced appetite independently of the food	Biting and aggressive when approached	Severe response: escape reaction
3	//	Continuous pacing in the box		//	No appetite	Stay immobile and disinterested	//

** items not assessed at extubation and sternal recumbency; * items not assessed at extubation.

Four animals (three males and one female) showed signs of acute pain and required rescue analgesia (maximum score of 8 for the males and 9 for the female) at Post MI. Pain peak occurred three hours after sternal recumbency (two males and one female) and one hour after sternal recumbency (one male). The four minipigs responded to the administration of rescue analgesia (morphine 0.2 mg/kg IM) and no further treatment was needed.

**Table 3:** Behavioural items recorded before the study endpoint (Post MI– endpoint). The score was recorded in a single evaluation 42 ± 3 days after myocardial infarction induction*.*

	Appearance	Lying and restlessness	Food interest	Aggression with comates	Isolation	Agitation at human approach	Desynchronization	Response to touch (neck, head, thorax and forelimbs)
0	Normal appearance	Normal lying	Normal appetite	Friendly	Look actively for interactions	Curious, interactive, may vocalise	Synchronized with mates	No response
1	Salivation	Lying guarding one part of the body/moves without external stimulation	Reduced appetite, eat special/ tasty food	Moves away	Not interested in interactions	Moves away when approached	//	Mild response: the animal looks uncomfortable and worried; retraction of the interested body part can be present
2	//	Move often/ poor wake times	Reduced appetite independently of the food	Biting and aggressive when approached by other mates	//	Biting and aggressive when approached	Desynchronized with mates	Severe response: escape reaction
3	//	Continuous pacing in the box	No appetite	No aggressions because of immobility	//	Stay immobile and disinterested	//	//

In these four animals, abnormalities of the following behaviours were recorded:

- Lying and restlessness: 4/4 minipigs
- Tremors and Spasms: 4/4 minipigs
- Vocalisation: 3/4 minipigs
- Food interest: 4/4 minipigs
- Agitation at human approach: 2/4 minipigs
- Response to touch: 1/4 minipig

Percentage of animals (whole sample, n = 24) showing or not showing the behavioural items considered at Post MI (independently from reaching the cut-off score for rescue analgesia administration) are reported in Table 4.

**Table 4:** Behavioural items included in the pain score showed by all minipigs at Post MI.

Parameter	Score	Percentage (%)
Appearance	0	100
	1	0
Lying and restlessness	0	45.83
	1	16.67
	2	29.17
	3	8.33
Tremors and spasms	0	45.83
	1	4.17
	2	50
Vocalisation	0	79.17
	1	20.38
Food interest	0	45.83
	1	4.17
	2	4.17
	3	45.83
Agitation at human approach	0	75
	1	25
Response to touch	0	83.33
	1	16.67

Number of animals reported as percentage (%).

In none of them, physiological parameters were modified. In two cases, invasive blood pressure was not recorded due to non-functional arterial catheter. Only one animal had a score of 1 for an increase of heart rate above 20% of its baseline.

The following correlations between MT and TT with behavioural items showed statistical significance:

- The decrease in TT from Post MI to Pre MI was associated with a lower likelihood of “Lying and restlessness” (OR = 0.52, p = 0.03) in females.
- The decrease in TT from Post MI to Post MI-endpoint and Pre MI to Post MI-endpoint was associated with a lower likelihood of losing appetite (parameter “Food interest”) (OR = 0.69, p = 0.039, OR = 0.74, p = 0.04, respectively)
- The decrease in TT from Pre MI to Post MI- endpoint is associated with a higher likelihood of tremors/spasms (OR = 1.38, p = 0.048).

After correcting for multiple testing, all the correlations lost statistically significance. Details are reported in supplementary file S7.

No animals showed abnormalities of the behavioural items at Post MI endpoint.

#### Cardiac biomarker and cytokines

Values of cardiac Troponin I (cTn-I), Tumour Necrosis Factor Alpha (TNFα), interleukin 6 (IL6) and interleukin 1 beta (IL1β) are reported in supplementary file S8.

**Fig. 3:**
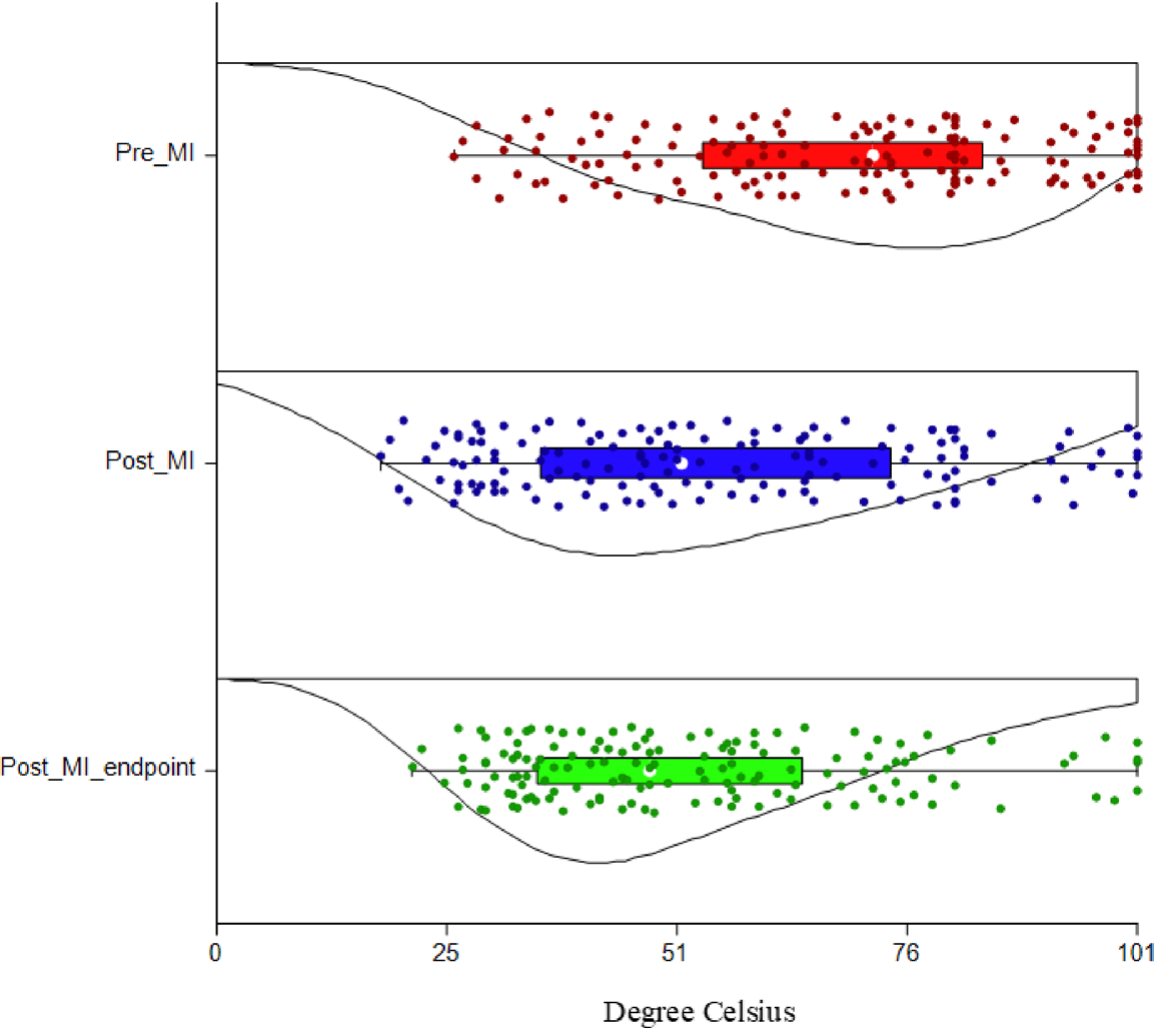
Box plots showing modifications in Troponin I detected at different days (Pre MI, Post MI and Post MI-endpoint) for the whole samples (n = 24) (**A**) and for each sex (11 females, 13 males) (**B**) (f: females, m: males) pg/ml: picograms/milliliter * Statistically significant difference compared to Pre MI; ** Statistically significant difference compared to Post MI.

In two animals (one at Pre MI and one at Post MI-endpoint) cTn-I was not quantified due to error in the analysis of the sample. Cardiac Tn-I significantly increased at Post MI compared to Pre MI (p < 0.001) and decreased at Post MI-endpoint compared to Post MI (p < 0.001) for both the whole sample (p < 0.001) and for both females (p < 0.001) and males (p < 0.001) (Fig. 3).

The IL1β decreased significantly at Post MI compared to Pre MI (p < 0.001) for the whole sample. When sex was considered, females did not show any statistically significant difference (p = 0.5). On the contrary, males showed a statistically significant decrease at Post MI compared to Pre MI (p < 0.001) and to Post MI-endpoint (p < 0.001).

No differences were found for both TNFα and IL6, both in the whole sample (p = 0.22 and p = 0.47, respectively) and when females (p = 0.94 and p = 0.28, respectively) and males (p = 0.67 and p = 0.78, respectively) were analysed separately.

Weak positive correlation between IL6 and TT at Pre MI (rho = 0.46, p = 0.025) and IL1β and TT at Pre MI (rho = 0.61, p = 0.028) were found. No other statistically significant correlations were present. Moreover, after correcting for multiple testing, all the correlations lost statistical significance. Further information is present in supplementary file S9.

- Physiological Parameters

Descriptive statistics for heart rate and respiratory rate is reported in supplementary file S10 (Supplementary table S10 and Supplementary fig S10).

Heart rate showed a statistically significant decrease at Post MI compared to Pre MI in the whole sample (p = 0.0046) and in females (p = 0.0038). On the contrary, no differences in heart rate were found in males only (p = 0.33). Respiratory rate increased (p = 0.005) at Post MI-endpoint compared to Post MI in the whole sample (p = 0.0058). When females were analysed separately, a statistically significant decrease at Post MI compared to Pre MI, and a statistically significant increase at Post MI endpoint compared to Post MI (p = 0.001) was found. No differences were found in males (p = 0.54).

- Feasibility scores

No animals had to be excluded from the QST due to lack of feasibility.

No differences were found at each day, both in the whole sample (MT: p = 0.39, TT p = 0.43) and when females and males were analysed separately (MT: females p = 0.76, males p = 0.61; TT: females p = 1, males p = 0.29). Histograms showing distribution of the feasibility score over days are presented in the Supplementary file S11 (Supplementary figs S8-S12). No correlations between feasibility score and median of MT and TT were found. Details are reported in supplementary file S12.

## Discussion

In the present study four minipigs (17%) showed moderate acute pain that required analgesia, while twenty minipigs (83%) showed no to mild acute pain. No minipigs showed behaviour alterations on the longer term and at the study endpoint. Mechanical and thermal thresholds decreased at Post MI and Post MI-endpoint. However, this decrease did not correlate with behavioural alterations leaving the door open to different interpretations of the recorded data. Sporadic and sparse responses to Von Frey were obtained in this study at Post MI and Post MI-endpoint, suggesting that no allodynia developed in minipigs following MI. Troponin I showed a large increase at Post MI, confirming myocardial damage, the degree of which did not correlate with the QST modifications recorded on the same day. Cytokines tended to decrease after myocardial infarction, and their plasma concentration did not correlate with QST.

The Swiss Commission for Ethics in Animal Experiments allocates models of myocardial infarction in the group of experiments with the highest prospective severity degree, which implies medium to long-term moderate or severe pain, suffering, injury, fear or severe impairment of the general health status of the animals. Our findings suggest that it is possible to refine this model and limit the duration and the intensity of pain. We think that the balanced anaesthesia protocol, the individualised acute pain treatment and the minimally invasive interventional procedure to create the infarction are pivotal to achieve this goal. Humans experiencing spontaneous MI reported chest pain in the following 24 h following PCI and can develop chronic pain^47,48^ . Minipigs underwent MI and reperfusion under controlled conditions and in general anaesthesia. Although chronic pain can occur despite the use of balanced anaesthesia and analgesia, due to many subjective and environmental related factors^49^, pain linked to ischaemia–reperfusion injuries is typically inflammatory. Indeed stopping and restarting vascularisation in the heart is known to cause nociception and pain due to the release of pro-inflammatory mediators^50^. Evidences suggested that proinflammatory cytokines, such as TNFα, IL6 and IL1β, can be used as potential pain biomarkers^39,42,51,52^ and that TNFα and IL1β can enhance pain transmission signals by lowering the activation threshold of nociceptors^53,54^.

Troponin I increased significantly at Post MI compared to Pre MI and then returned at baseline concentration at the study endpoint. Its increase is indicative of presence of myocardial damage and successful MI induction, despite reperfusion was carried out. This is in line with previous literature: in humans, troponin I can be still elevated after successful PCI^15,16^, and in pigs the same applied after left anterior descending (LAD) coronary artery ischaemia and reperfusion. As hypothesised, troponin changes did not correlate with modification of MT and TT and with any behaviours associated with pain, aligning with human findings that more extensive cardiac damage does not reflect more severe pain^55^ . Mechanical and thermal thresholds decreased at Post MI and Post MI-endpoint compared to Pre MI. This might be interpreted as modification of pain perception and described as somatic hyperalgesia deriving from visceral pain.

Pain arising from the heart following an ischaemic event is classified as visceral pain, which refer to somatic areas (referred pain)^12^. Referred pain is explained by the convergence projection theory: somatic and cardiac afferents converge into the same tract of the spinal cord (lamina I), making them indistinguishable by higher centres^56^. In humans, referred pain due to MI is mainly reported at the level of the chest, forearm, neck and epigastric region^12^, which in some patients can last up one year after the occurrence of the ischaemic event and the resolutive PCI^16^. In rats following experimentally induced MI, referred somatic hypersensitivity was observed in left chest, forelimb, and upper back, and was shown to be present also at 28 days post insult^57^. In our case, a decrease of mechanical thresholds was detected until Post MI-endpoint (day 42 ± 3). In the present study, no correlation was found between the decrease in QST thresholds and the occurrence of behaviours associated with pain. Therefore, we remain conscious in interpreting this finding. In human medicine, it has been reported that patients experiencing pain due to MI showed lower thermal thresholds than subjects having a silent MI (no pain). While QST are used to assess hyperalgesia, i.e., an enhanced pain response to a stimulus that typically provokes pain^58^, behavioural aspects are mainly used to evaluate pain. Pain is indeed a complex, multidimensional experience, encompassing both sensory and emotional-cognitive components^21^. The evaluation of both components is essential for a comprehensive evaluation of pain^21,59^. Assessing mechanical and thermal thresholds in animals remains challenging due to their nonverbal nature. While the assessment in humans can rely on the patients’ communication, in animals it must rely on evoked behavioural responses to stimuli. This poses the challenge to distinguish between genuine aversion to stimulation, and reactions to discomfort or nuisance. To enhance the likelihood of collecting reliable data, we developed a feasibility score, based on our previous experience in minipigs^60^. The score served two purposes: (1) to exclude animals that exhibited uncooperating behaviour, preventing a correct thresholds interpretation during the procedure, and (2) to evaluate whether the level of feasibility influenced per se the thresholds. No animals were excluded, and no correlation was found between thresholds and feasibility scores, suggesting that our measurements detected the effect of the disease rather than variations in animals’ cooperation. However, the absence of a control group prevents us from a definitive statement about the value of these findings.

Mechanical and thermal thresholds did not exhibit the same extent of decrease at Post MI-endpoint. A possible explanation for this discrepancy is the type of stimulus used. Whether thermal stimuli could detect a deep somatic pain has been previously questioned^61^. Furthermore, although in different environmental conditions, thermal thresholds showed a poor reliability in minipigs^60^. Whether the heat stimulus is able to reach cutaneous nociceptors has also been questioned in horses^27^. Although minipigs and pigs’ skin has been shown to resemble human skin^62^, differences in the amount of subcutaneous fat layer and skin thickness exist^63^. Thus, it remains unclear whether the temperature recorded at the level of the skin surface accurately reflects the temperature that reaches the cutaneous thermal nociceptors. Our device has an upper limit temperature of 55 °C which has been previously set to avoid burning of the skin. It is unclear whether this cut-off could have influenced our results. Moreover, contact heat pain delivered at slow rate, as done in the present study, has been shown to induce prevalently C fibers activation^64^. Use of thermal stimuli of different nature, such as CO_2_ laser might have led to different results due to its ability to prevalently activate Aδ fibers^65^.

Physiological parameters (heart rate, respiratory rate and invasive blood pressure) did not contribute to any increase in pain scores, in none of the days. The assessment of heart rate and blood pressure variations to detect pain and nociception is still widely used both in awake and anaesthetised pigs^66,67^. In a context like MI induction, however, we should question their utility considering that modifications of heart rate and arterial blood pressure are most likely brought about by the condition per se and not by pain. In the present study, heart rate recorded during QST assessment (15–20 h after sternal recumbency), showed a statistically significant decrease compared to Pre MI. Sinus bradycardia is a common finding in humans experiencing MI and tends to resolve in 24 h^68^.

In the present study, a sub analysis to look at sex-related differences was performed. While females drove the results toward a decrease in MT at Post MI, males drove the results of both MT and TT toward a decrease at Post MI-endpoint. We might suggest that males might be more prone to development of long-lasting hypersensitivity than females. Sex-related differences are well known in the field of pain and cardiovascular diseases^69,70^ and oestrogen role in modulating pain sensitivity has also been demonstrated^71,72^. However, based on the current literature, it is hard to give an explanation on the results of this study^71,73^. A sex bias in studies involving animals has been reported, in several species: male subjects have mainly been used in the past, and only recently attention to the topic has been raised^69,74^. Further investigations should be conducted to explore the influence of sex on nociception, hyperalgesia and pain in animal species.

This study has some limitations that must be acknowledged. 1) Blood samples for determination of troponin I were collected ten days before the study endpoint. However, since no clinical changes were found in any animal, and signs of heart insufficiency were not detected, we consider unlikely that any clinically relevant modification in troponin I levels occurred at the study endpoint. 2) We opted for a purpose-built score based on modifications in physiological variables and behavioural items previously correlated with pain. Our rationale was that minipigs enrolled in the present trial were adult animals differently from the previous studies and the only validated scale for the species targeted a husbandry procedure (i.e., castration in piglets)^36,37^. The cut-off for pain treatment was not validated. 3) All the minipigs enrolled in these trials underwent MI induction, and no control group was present. This limitation is intrinsic in the study design. 4) No power calculation was performed a priori for the present trial, since it was done for the preclinical study. 5) Quantitative sensory testing and pain score were conducted no later than 42 ± 3 days after MI induction. This time point was the last available before the termination of the preclinical study in which the minipigs were enrolled. We cannot provide any data on modifications of QST or pain development in a longer period, which precludes us to give answers regarding the evolution of local hyperalgesia.

## Conclusions and outlook

Cardiovascular models, such as MI, are deemed to impose a severe burden on the animals. Our results underline that model refinement significantly contributes to limiting pain and suffering. Prompt and individualised treatment of acute pain prevented the development of sub-acute pain. It is possible that myocardial infarction leads to somatic hyperalgesia; however, the pain thresholds do not correlate with either behavioural signs of pain or the severity of the infarction. Further studies on specific behavioural scales for Göttingen minipigs are warranted, and the scale developed for our trial could serve as a basis. Investigating pain in experimental models remains challenging due to the non-verbal nature of the animals, but it can provide critical insights into pain pathophysiology, which are essential for scientific advancements in both human and veterinary medicine.

## Materials and methods

### Animals

Twenty-seven Göttingen Minipigs, 12 females and 15 males, aged 15.6 ± 2.5 months and weighing 30.4 ± 4 kg were enrolled in the study. All the minipigs of this study were purchased from their official breeder (Ellegaard Göttingen Minipigs, Dalmose, Denmark) for a preclinical study investigating the effect of an antifibrotic therapy following MI. The animals were transported to Switzerland, where they were hosted in groups of six to ten in an 18 m^2^ pen with a 50 m^2^ outdoor area located on a farm. Water was available ad libitum, and food (Minipigs Maintenance Standard, Kliba Nafag, Switzerland) was provided twice per day (800 g/minipig/day). Enrichment was guaranteed with straw and toys (hanging ropes and balls). At least two weeks of acclimatisation were allowed before starting the trial. During this time, the animals were accustomed to the environment, the presence of researchers and caretakers, handling and manipulations, positioning in the sling and clinical examinations, and they learned simple tasks as moving onto a scale for body weight measurement and approaching humans without fear. Special food was offered as reward (apple juice or mousse). Physiological and behavioural evaluations were carried out at least three time per week before the beginning of the trial. Further details on housing conditions have been previously published^60^ and details on the score used to assess their wellbeing is reported in supplementary file S13.

At least 24 h before MI induction, minipigs were transported in groups of two from the hosting farm to the Experimental Surgery Facility (ESF) of the University of Bern. There, they were hosted together in the large animal intensive care unit in a box (length: 2.07 × width: 1.68 × height: 2.50 square meters) until MI induction.

After the procedure, the animals were hosted into two different boxes as still instrumented with arterial catheter and port-catheter needle/venous access; however, they could keep auditory and visual contact with the co-mate (barrier between the two stables is provided with holes). All the minipigs were kept under veterinary observation for at least 15 h after reaching sternal recumbency during anaesthesia recovery, and they were moved back to the farm only when deemed pain free and clinically stable.

Following MI induction, minipigs were kept at the farm until the study endpoint (day 42 ± 3 after MI). Veterinary check (including evaluation of both behavioural and physiological parameters) was performed daily from post-operative (PO) day 1 to PO day 7, and from PO day 8 to PO day 42 ± 3 at least three times per week. In particular, the animals were checked for pain and any other secondary pathological signs such as diarrhoea, nausea, vomiting or signs of heart insufficiency. Meloxicam (Metacam, Boehringer Ingelheim Schweiz GmbH) 0.4 mg/kg (once daily) and Esomeprazole (Nexium 40 mg, Grünenthal Pharma AG, Switzerland) 1 mg/kg (twice daily) were administered orally from PO day 1 to PO day 3. If pain was detected according to a purpose-built pain scale (Table 3 and supplementary file S14), rescue analgesia was administered. Body weight was checked daily until PO day 3, and then once per week until the study endpoint.

On the day of euthanasia (study endpoint, PO day 42 ± 3), the minipigs were transported from the farm to the ESF where they were anesthetised for undergoing cardiac magnetic resonance imaging (MRI)^75^. Following preanesthetic clinical examination and sedation, they were transported to the TIC-SITEM of the University of Bern, where preparation for general anaesthesia was completed and the scan was carried out. At the end of the procedure, minipigs were euthanized under general anaesthesia with an intravenous overdose of pentobarbital (100 mg/kg).

The study was designed as a prospective cohort study. It was reviewed and approved by the Committee for Animal Experiments of the Canton of Bern, Switzerland (national number: 33492) and all methods were performed in accordance with the relevant guidelines and regulations. In particular, for reporting methods and results, the ARRIVE guidelines were strictly followed^76^.

### Course of the experiment

All the data were recorded at the Large Animal Intensive Care Unit of the ESF, University of Bern.

The study was carried out between September 2022 and December 2023.

Quantitative sensory testing data and physiological parameters (heart rate, respiratory rate) were collected at three days:

1. *Pre MI*: the day before MI induction
2. *Post MI*: 15–20 h after sternal recumbency (SR)
3. *Post MI- endpoint*: study endpoint (PO day 42 ± 3)

Pain scores were performed postoperatively at days:

1. *Post MI*: multiple evaluations performed at different time points after the end of the procedure (MI induction):

- Extubation
- Sternal recumbency (SR)
- 15 min after SR
- 1 h after SR
- 3 h after SR
- 6 h after SR
- 9 h after SR
- 12 h after SR

2) *Post MI- endpoint:* single evaluation performed at the study endpoint (PO day 42 ± 3).

Blood samples for troponin I and cytokines were collected at days:

1. *Pre MI*: early in the morning on day of MI induction, before any manipulation
2. *Post MI*: the morning after MI induction, 13–18 h after SR
3. *Post MI- endpoint*: around one week before the study endpoint (PO day 35 ± 2)

### Quantitative sensory testing and physiological parameters assessment

Quantitative sensory testing was carried out at the ESF where conditions of temperature (22° C) and humidity (40–60%) were maintained stable. Minipigs were placed in a sling, to which they were accustomed and, to reduce stress, auditory and olfactory contact with a co-mate was guaranteed. Recording of QST thresholds was always performed in animals recently fed to increase cooperation.

At the beginning of each session (Pre MI, Post MI and Post MI– endpoint) physiological parameters, namely heart rate and respiratory rate were recorded.

Development of allodynia was then assessed using von Frey filaments (ranging from 1 to 300 g/cm^2^).

Afterwards, mechanical and thermal thresholds (MT and TT, respectively) were tested, and their order was assigned based on a randomisation into blocks (www.randomization.com).

The exact methodologies applied for MT, TT and von Frey has been previously described^60^. Briefly, TT were determined using a custom-made thermal stimulator with a 1 cm^2^ contact area probe (cut-off stimulation 55 degrees Celsius (°C), start stimulation probe temperature: 20° C). Mechanical thresholds were determined with a handheld mechanical algometer (FPN 100–Algometer, Wagner Instruments, Greenwich, Connecticut, USA) equipped with a 1 cm^2^ rubber tip. However, in this trial, based on results from our previous trial^60^ the cut-off for mechanical thresholds was set at 100 Newton (equivalent to 1000 Kilopascals).

Thresholds to mechanical (algometer and Von Frey) and thermal stimulations were defined at the value measured when the animals showed the following evoked behavioural responses:

- withdrawal of the tested area (e.g. limb lifted)
- vocalisation and watching in the direction of the tested area
- muscle twitch at the level of the tested area with head turned in that direction
- escape reactions (rapid body movement to move away from the source of the stimulation)

Thresholds were recorded in body areas where pain due to MI is typically referred in humans^12^, namely:

- Forearm, left and right (LF, RF)
- Chest, left and right (LC, RC)
- Neck, left and right (LN, RN)

Further details on the tested site have been previously published^60^.

Sites were tested in a random order; if the sites were tested twice, the same order was maintained.

Quantitative sensory testing was always carried out by the same operator (MP), who was familiar with the animals and the technique, to reduce detection bias. Thresholds values were recorded on the on-purpose made experimental sheet by another operator (CP, LGGC or DC) to reduce observer bias.

A feasibility score, slightly modified from a previous published version ^60^, was used to assess the cooperation of each minipigs to both MT and TT assessment at each day for both MT and TT. Animals showing a feasibility score of 3 were excluded from the study. Details about are reported in supplementary file S15.

At the end of the recording session, minipigs were rewarded with apple juice or mousse, and moved back into their box.

### Pain score assessment

Assessment was performed when the animals were housed individually at Post MI (at each of the abovementioned time points) and when they were in presence of a co-mate at Post MI-endpoint. Pain scoring was performed by MP, CP and LGGC.

Both behavioural and physiological parameters were considered.

Heart rate and respiratory rate were assessed at Post MI and Post MI- endpoint; arterial blood pressure (Post MI only) was assessed only at Post MI. The score was allotted as follows:

*Heart rate and respiratory rate* (0–2):

1. Heart rate and respiratory comparable to baseline (increase ≤ 20% from baseline)
2. Moderate increase of only one parameter (either heart rate or respiratory rate), between 20 and 30% of the normal range
3. Severe increase (more than 30%) of only one or both parameters.

Baseline were considered values recorded during the pre-anaesthetic examination.

*Arterial blood pressure* (0–2):

- Systolic arterial pressure (SAP) within the normal range of variation (110- 160 mmHg)
- Moderate increase of the SAP (161 < x < 200 mmHg)
- Severe increase of the SAP (> 200 mmHg)

Behaviours assessed at each day are reported in Tables 2 (Post MI) and 3 (Post MI- endpoint). For each item, a score ranging from 0 to 3 was assigned. Behavioural items associated with pain included in the two scores were based on previous publication on the topic^37,66,77,78^.

The items “food interest” and “agitation at human approach” were not assessed at extubation, whereas “appearance” and “lying and restlessness” were not assessed at extubation and SR (Post MI).

Rescue analgesia was administered in case of:

- Post MI: score ≥ 4/11 at extubation; score ≥ 4/17 at SR; score ≥ 7/21 from SR to 12 h after SR.
- Post MI- endpoint: score ≥ 5/22 at Post MI- endpoint.

Further details on both pain scores are reported in supplementary file S16 (Post MI) and S14 (Post MI-endpoint).

### Blood samples collection

Venous blood was collected from the jugular port-catheter implanted in the facility of origin. The minipigs were positioned into the sling and the operator performed the procedure under sterile conditions. The skin overlying the port-catheter was shaved, cleaned with Povidone-Iodine soap (Betadine soap, Provet AG, Switzerland) and water and then disinfected with sterile wipes soaked with Povidone-Iodine and Alcohol (Betaseptic, Provet AG, Switzerland). Once the skin was dry, a purpose made needle (20 Gauge × 25 mm, Bard Access System, Inc, USA) was connected to a 10 ml sterile syringe (PosiFlush SP, BD, Switzerland) and the skin was punctured. A volume of 3 ml of blood was withdrawn and discarded before blood collection. After blood collection, the catheter was flushed with 10 ml of sodium chloride followed by 3 ml of sodium heparin (Heparin Bichsel, 100 IU/ml, Bichsel, Switzerland) to avoid clotting.

In case of no implanted port-catheter or malfunctioning, arterial blood was collected using a 22 Gauge arterial catheter (BD Venflon Pro Safety; BD, Switzerland) positioned on the coccygeal artery.

### Troponin I and cytokines assay

Blood was collected into EDTA tubes (S-Monovette, Sarstedt AG, Germany), centrifuged (2,000 × g for 15 min at 4 °C) and plasma was then moved in 500 µl labelled cryotubes and stored at -80 °C.

Details about the technique used for troponin I and cytokines quantification have been previously published^79^. Briefly, myocardial damage marker cardiac troponin I (cTn-I) and the inflammation biomarkers were measured using a multiplex xMAP technology (Luminex) assay according to a custom-made protocol. Fluorochrome microbeads (Luminex) were coupled with respective capture antibodies using the Bio-Plex amine coupling kit (Bio-Rad). Coupled beads were then incubated with pig plasma samples, followed by biotinylated detection antibodies and Streptavidin-PE (Bio-Rad). Measurement and data analysis were performed with a Flexmap 3D reader and the Bio-Plex Manager software version 6.1 (Bio-Rad).

Cytokines analysed for this study were TNFα, IL-1β and IL-6.

Operator in charge of blood sample analysis (AD) was blinded to results related to QST and pain scores.

### Myocardial infarction induction

#### Anaesthesia

Minipigs were placed in a sling, a port needle was inserted in the jugular port catheter and venous blood was collected for blood work. Thereafter, minipigs were sedated with 0.2 mg/kg morphine (Morphine HCl 2 mg/ml, Sintetica AG) 10 mg/kg ketamine (Narketan 100 mg/ml, Vetoquinol AG) and 15 mcg/kg dexmedetomine (Dexdomitor 0.5 mg/ml, Provet AG) mixed in the same syringe and injected intramuscularly (IM). If sedation was deemed inadequate 15 min after injection, 0.2 mg/kg midazolam (Dormicum 50 mg/10 ml, Roche) was injected IM.

Once sedated, minipigs were moved on a table, supplemented with oxygen through a face mask and monitored with a pulse-oximeter. An auricular vein was cannulated and fluid administration using Ringer Lactate’s solution was started and continued for the whole procedure at 5 ml/kg/h. Anaesthesia was inducted with ketamine 1 mg/kg and propofol (Propofol 10 mg/ml, Fresenius Kabi, Switzerland) to effect (1–3 mg/kg), injected intravenously (IV). Following tracheal intubation, the endotracheal tube was connected to a re-breathing system. Anaesthesia was deepened and maintained with sevoflurane in oxygen and compressed air. End tidal sevoflurane was adjusted to guarantee adequate depth of anaesthesia up to its MAC (2.2%)^80^. Spontaneous breathing was supported, or positive pressure ventilation was initiated using a positive end expiratory pressure (PEEP) of 5 cmH_2_O, and tidal volume (TV) of 8–12 mL/kg body weight, targeting a PaCO_2_ of 40–45 mmHg, if spontaneous breathing did not guarantee normocapnia. Amoxicillin-clavulanic acid (Clamoxyl 20, GlaxoSmithKline Pharmaceutical s.a./n.v.) 20 mg/kg was administered IV. In the operation room, unless bradycardia was present (HR < 50 bpm) dexmedetomidine continuous rate infusion (CRI) was started at a dose range of 1–5 mcg/kg/h.

During general anaesthesia, minipigs were continuously monitored for HR, RR, SpO_2_, capnography, invasive blood pressure, oesophageal temperature, inspired and expired fraction of gases (air, oxygen), central venous pressure and electroencephalographic activity (EEG) (Sedline, Masimo Corp., CA, USA). A temperature of (35–36° C) was targeted along the whole intervention and maintained with the help of a forced air device (Mistral-air forced air warming system) (84). Before LAD catheterisation, amiodarone 5 mg/kg (Cordarone 50 ml/ml, Sanofi-Aventis) was administered over 1 h, and continuous rate infusion (CRI) of lidocaine 30 μg/kg/min (Lidocaine 1%, Streuli Pharma AG) was started. A mean arterial blood pressure (MAP) of 65 mmHg was targeted, and hypotension was treated with Dobutamine 1–5 mcg/kg/min (Dobutrex 5 mg/ml, Teva Pharma AG) IV titrated to effect, and calcium gluconate 0.2–0.3 mmol/kg/h (Calciumgluconat 10%, B.Braun) IV. Noradrenaline 0.1–0.5 mcg/kg/minute (Noradrenaline 1 mg/mL, Sintetica) was administered if other inotropes could not resolve the hypotension. Cardiovascular relevant arrhythmias were treated chemically or electrically, according to the type of arrhythmia. In presence of nociception, rescue analgesia was provided with 3–5 mcg/kg fentanyl bolus (Fentanyl 0.5 mg/10 mL, Sintetica) or 1 mg/kg ketamine IV. Before LAD catheterisation, activated clotting time (ACT) was assessed and a bolus of heparin (Liquemin 5000 I.U/5 ml, Drossapharm AG) 80 I.U./kg was administered, followed by heparin CRI. Its rate was adjusted by targeting ACT values of two–three times its baseline value to prevent the risk of wire and sheath thrombosis. Cardiopulmonary resuscitation (CPR) was carried out in case of sudden cardiac arrest or ventricular fibrillation, based on guidelines for small animals (https://recoverinitiative.org/2012-guidelines); if the minipigs were unresponsive to any procedures 30 min after starting CPR, death was declared.

At the end of procedure, minipigs were weaned from the ventilator and transported in the recovery box once able to breathe spontaneously. Tracheal extubation was performed when minipigs showed return of swallowing reflex. Post-operative sedation (dexmedetomidine 0.5–3 mcg/kg/hour) and fluid therapy (Ringer Lactate 3–5 ml/kg/h) were administered from extubation to sternal recumbency. Lidocaine CRI was continued during the recovery in presence of cardiac arrhythmias and discontinued if sinus rhythm and blood pressure were stable. The minipigs were monitored for HR, SpO_2_ and invasive blood pressure through a multimodular monitor (S/5 Critical Care Monitor; Datex-Ohmeda, GE Healthcare) until able to stand and walk. Furthermore, minipigs received meloxicam (Metacam, Boehringer Ingelheim Schweiz GmbH) 0.4 mg/kg IV, furosemide (Lasix 20 mg/2 ml, Sanofi-Aventis) 2.5 mg/kg IV and nitro-glycerine (Nitroglycerin 0.1 mg/ml, Sintetica SA) 0.2 mg IV over 30 min.

#### Myocardial infarction induction procedure

**Fig. 4:**
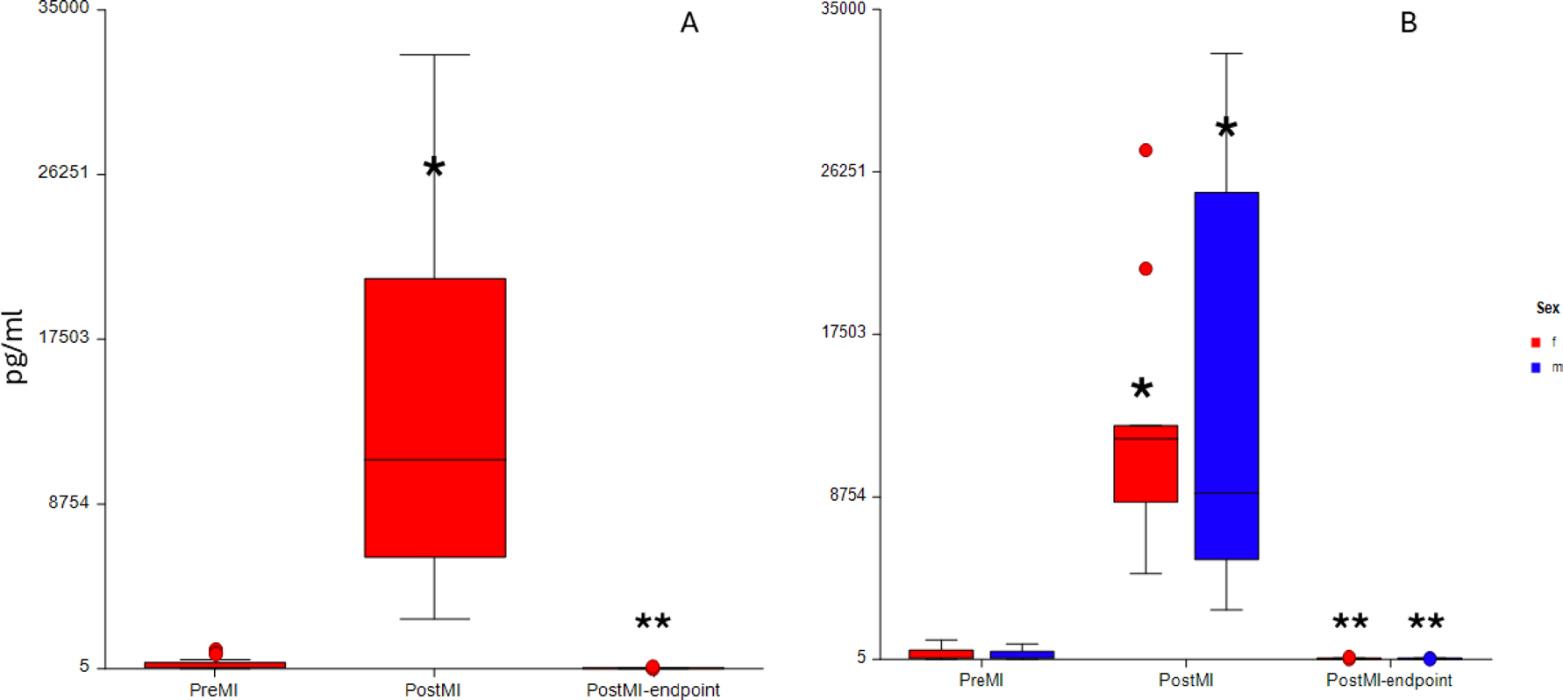
Fluoroscopic image of the heart taken during the procedure. Left anterior descending coronary artery has been occluded with a balloon catheter during the ischaemic phase. Occlusion site is indicated with the blue arrow.

After preparation for general anaesthesia, the minipigs were moved in the Hybrid-Catheter Lab operation room (ACT-Inno AG, Inselspital) and placed on the angiographic table in dorsal recumbency. Right femoral artery was cannulated under ultrasound guidance with a modified Seldinger’s technique. A long 6 French arterial vascular sheath was then inserted in the vessel, and the sheath was sutured. Standard catheter shapes designed for human procedures (for instance, Judkins left and right) were used to allow selective access to the left the coronary artery. A work-horse coronary wire (Hi-Torque balanced Middleweight) was engaged in the distal LAD coronary artery. A balloon was then introduced and inflated in the middle part of the artery, leaving the first diagonal branch (or alternatively the intermediate branch) patent. Complete occlusion of the vessel was confirmed by coronary angiography with injection of contrast medium and the presence of ECG modifications (e.g. ST elevation). The balloon was left inflated for 90 min (ischaemic phase, Fig. 4) and then deflated to allow reperfusion of the LAD. The minipigs were maintained under general anaesthesia for 120 min during the reperfusion phase. Thereafter, all the procedural catheters were removed before recovery.

All the animals received two injections of the experimental antifibrotic treatment or placebo (based on a random assignation) few days following MI induction (PO day 3 and PO day 16), but no other medications currently in use in human patients following MI and PCI (e.g. beta blockers) were administered at any time.

### Statistical analysis

Statistical analysis was conducted with R (version 4.2.3) and NCSS Statistical Software. Data normality was assessed with the Shapiro Wilk test.

For MT and Von Frey, the arithmetic mean of the two recorded values, for each tested site, was calculated and used for further analysis. Furthermore, when the cut-off of stimulation was reached before obtaining a reaction of the animals, the values of 101 N and 56 °C was used for the statistical analysis for MT and TT, respectively.

Median and interquartile range (IQR [25^th^;75^th^]) of MT, TT and Von Frey were calculated, per day, for the whole sample and for females and males separately, *a)* independently from the tested site (thresholds coming from sites LC, RC, LN, RN, LF and RF were pooled together and treated as independent measurements) *b)* for each site independently.

Differences between two different days X and Y were evaluated by *a)* taking the median of paired differences between days (LC_dayX_-LC_dayY_, RC_dayX_-RC_dayY_,etc.) *b)* considering the influence of each tested site LC, RC etc. individually. A one-sample t-test was performed under the null hypothesis that there was no difference between the two days, both for the whole sample and for each sex. The p-value was calculated from the Student’s distribution, provided the Shapiro Wilk test reported a p value > 0.05; when not, permutation tests p values were considered instead. The reported effect size is the Cohen’s d coefficient.

Correlation between pain-related behaviours present in the pain scores and differences between days of medians of MT and TT and Von Frey, both for the whole sample and for each sex, were assessed using Ordinal Logistic Regression (R package polr) when at least 3 levels were available, and by Binomial Logistic Regression (R package glm) otherwise. Odds Ratio (OR) is reported. For ordinal logistic regression, the 2-tailed Wald z test was used to estimate the significance of the OR. In both cases, confidence intervals were calculated by using the normal distribution.

To analyse modifications of cTn-I, TNFα, IL6 and IL1β between days (both for the whole sample and for each sex), the Friedman Repeated Measures Analysis of Variance on Ranks followed by Bonferroni post-hoc test for pairwise comparison was used. Moreover, Spearman’s rank test was used to assess correlation between these variables and median of MT, TT and Von Frey for each day.

To assess modifications of physiological parameters (HR, RR) over days, the Friedman Repeated Measures Analysis of Variance on Ranks followed by Bonferroni post-hoc test for pairwise comparison was used. Analysis was conducted both for the whole sample and for each sex.

To evaluate modifications in feasibility scores at different days, the Chi-square test of homogeneity was performed, both for MT, TT and Von Frey and both for the whole sample and for each sex.

Adjusted p-values reflecting the false discovery rate are calculated following the Benjamin-Hochberg method^82^ and are applied separately on sets of tests performed at different time points or time differences.

## Supplementary Information

Below is the link to the electronic supplementary material.

Supplementary Material 1

Supplementary Material 2

Supplementary Material 3

Supplementary Material 4

Supplementary Material 5

Supplementary Material 6

Supplementary Material 7

Supplementary Material 8

Supplementary Material 9

Supplementary Material 10

Supplementary Material 11

Supplementary Material 12

Supplementary Material 13

Supplementary Material 14

Supplementary Material 15

Supplementary Material 16

## Data Availability

The datasets presented in this study can be found in the online repositories: DOI10.5281/zenodo.14003743
