# Supplementary material for: Unravelling pain in Göttingen Minipigs undergoing experimentally induced closed-chest myocardial infarction: a prospective cohort study
Source: Sci Rep. 2025 Oct 22;15:36934. doi: 10.1038/s41598-025-20920-y (PMC12546812; doi:10.1038/s41598-025-20920-y)
Supplement: Supplementary file 6 — Supplementary Material 6 [file 41598_2025_20920_MOESM6_ESM.docx]

|  | **PRE MI** | **POST MI** | **POST MI Endpoint** | **PRE MI** | **POST MI** | **POST MI Endpoint** | **PRE MI** | **POST MI** | **POST MI Endpoint** | **PRE MI** | **POST MI** | **POST MI Endpoint** | **PRE MI** | **POST MI** | | **POST MI Endpoint** | **PRE MI** | **POST MI** | **POST MI Endpoint** |
| --- | --- | --- | --- | --- | --- | --- | --- | --- | --- | --- | --- | --- | --- | --- | --- | --- | --- | --- | --- |
| **Minipig**  (sex) | **LF** | | | **RF** | | | **LC** | | | **RC** | | | **LN** | | | | **RN** | | |
| **1** (f) | no | no | no | no | no | no | no | no | no | no | no | no | no | no | no | | no | no | no |
| **2** (m) | no | no | no | no | no | no | no | no | no | no | no | no | no | no | no | | no | no | no |
| **3** (m) | no | no | no | no | no | no | no | no | no | no | no | no | no | no | no | | no | no | no |
| **4** (m) | no | no | no | no | no | no | no | no | no | no | no | no | no | no | no | | no | no | no |
| **5** (m) | no | no | no | no | no | no | no | no | no | no | no | no | no | no | no | | no | no | no |
| **6** (m) | no | no | no | no | no | no | no | 180 | no | no | no | no | no | no | no | | no | no | no |
| **7** (m) | no | no | no | no | no | no | no | no | no | no | no | no | no | no | no | | no | no | no |
| **8** (f) | no | no | no | no | no | no | no | no | no | no | no | no | no | no | no | | no | no | no |
| **9** (f) | no | no | no | no | no | no | 100 | no | no | no | no | no | no | no | no | | no | no | no |
| **10** (f) | no | no | no | no | no | no | no | no | no | no | 60 | no | no | no | no | | no | no | no |
| **11** (f) | no | no | no | no | no | no | 26 | no | no | no | no | no | no | no | no | | no | no | no |
| **12** (m) | no | no | no | no | no | no | no | no | no | no | no | no | no | no | no | | no | no | no |
| **13** (f) | no | 300 | no | no | no | no | no | no | no | no | no | no | no | no | no | | no | no | no |
| **14** (f) | no | no | no | no | no | no | no | no | no | no | no | no | no | no | no | | no | no | no |
| **15** (f) | no | no | no | no | no | no | no | no | no | no | no | no | no | no | no | | no | no | no |
| **16** (m) | no | no | no | no | no | no | no | no | no | no | no | no | no | no | no | | no | no | no |
| **17** (m) | no | no | no | no | no | no | no | no | no | no | no | no | no | no | no | | no | no | no |
| **18** (f) | no | no | no | no | no | no | no | no | no | no | no | no | no | no | no | | no | no | no |
| **19** (f) | no | no | no | no | no | no | no | no | no | no | no | no | no | no | no | | no | no | no |
| **20** (m) | no | no | no | no | no | no | no | no | no | no | no | no | no | no | no | | no | no | no |
| **21** (m) | no | no | no | no | no | no | no | no | no | no | no | no | no | no | no | | no | no | no |
| **22** (f) | no | no | no | no | no | no | no | no | no | no | no | no | no | no | no | | no | no | no |
| **23** (m) | no | no | no | no | no | no | no | no | no | no | no | no | no | no | no | | no | no | no |
| **24** (m) | no | no | no | no | no | no | no | 100 | no | no | no | no | no | no | no | | no | no | no |

**Supplementary file S1. Results of Von Frey testing, per each minipig, day** (Pre MI, Post MI and Post MI-endpoint) **and site** (LF: left forearm; RF: right forearm; LC: left chest; RC: right chest; LN: left neck; RN: right neck). f: females; m: males. Tested filaments: 1, 2, 4, 8, 10, 15, 26, 60, 100, 180, 300 g/cm^2^.
