# Supplementary material for: Unravelling pain in Göttingen Minipigs undergoing experimentally induced closed-chest myocardial infarction: a prospective cohort study
Source: Sci Rep. 2025 Oct 22;15:36934. doi: 10.1038/s41598-025-20920-y (PMC12546812; doi:10.1038/s41598-025-20920-y)
Supplement: Supplementary file 8 — Supplementary Material 8 [file 41598_2025_20920_MOESM8_ESM.docx]

**Supplementary file S6.** Side-related modifications of mechanical thresholds (MT) and thermal thresholds (TT) for each day (Pre-MI, Post MI and Post MI- endpoint) and sex.

| Day | Sex | QST | Site (p value) | D coefficient (effect size) |
| --- | --- | --- | --- | --- |
| Pre MI – Post MI | All (n=24) | MT | RN (p< 0.001)  RC (p= 0.001)  LN (p= 0.007)  LF (p= 0.01)  *RF (p= 0.04)* | RN= -0.9  RC= -0.71  LN= -0.6  LF= -0.56  *RF= -0.45* |
|  |  | TT | LC (p= 0.0004)  RN (p= 0.007)  *LN (p= 0.048)* | LC= -0.85  RN= -0.6  *LN= -0.42* |
|  | Females (n=11) | MT | RN (p= 0.001)  LF (p= 0.004)  *RF (p= 0.03)*  *RC (p= 0.03)* | RN= -1.3  LF= -1.14  *RF= -0.78*  *RC= -0.75* |
|  |  | TT | *RN (p= 0.014)*  *LC (p= 0.02)* | *RN= -0.89*  *LC= -0.81* |
|  | Males (n=13) | MT | *RC (p= 0.038)*  *LN (p= 0.04)*  *RN (p= 0.04)* | *RC= -0.64*  *LN= -0.63*  *RN= -0.62* |
|  |  | TT | LC (p= 0.008) | LC= -0.87 |
| Pre MI – Post MI- endpoint | All (n=24) | MT | RN (p< 0.001)  LN (p< 0.001)  RC (p= 0.0051)  LC (p= 0.0084)  LF (p= 0.013)  RF (p= 0.029) | RN= 1.26  LN= 0.79  RC= 0.63  LC= 0.58  LF= 0.54  RF= 0.47 |
|  |  | TT | *RN (p= 0.026)*  *LN (p= 0.035)* | *RN= 0.48*  *LN= 0.45* |
|  | Females  (n=11) | MT | RN (p= 0.0037)  *RC (p= 0.020)* | RN= 1.13  *RC= 0.83* |
|  |  | TT | // | // |
|  | Males (n=13) | MT | LN (p< 0.001)  RN (p< 0.001)  LF (p= 0.010)  *LC (p= 0.026)* | LN= 1.42  RN= 1.4  LF= 0.84  *LC= 0.7* |
|  |  | TT | LC (p= 0.002)  RN (p= 0.01)  *LN (p= 0.03)* | LC= 0.95  RN= 0.79  *LN= 0.65* |
| Post MI – Post MI- endpoint | All (n=24) | MT | // | // |
|  |  | TT | // | // |
|  | Females (n=11) | MT | *LF (p= 0.02)* | *LF= -0.7* |
|  |  | TT | // | // |
|  | Males (n=13) | MT | *RN (p= 0.03)* | *RN= 0.64* |
|  |  | TT | *RF (p= 0.01)*  *RC (p= 0.01)* | *RF= 0.84*  *RC= 0.82* |

QST: quantitative sensory testing; LF: left forearm; RF: right forearm; LC: left chest; RC: right chest; LN: left neck; RN: right neck. Values in italics are those for which the adjusted p value was > 0.05 after multiple tests correction.
