# Supplementary material for: Unravelling pain in Göttingen Minipigs undergoing experimentally induced closed-chest myocardial infarction: a prospective cohort study
Source: Sci Rep. 2025 Oct 22;15:36934. doi: 10.1038/s41598-025-20920-y (PMC12546812; doi:10.1038/s41598-025-20920-y)
Supplement: Supplementary file 9 — Supplementary Material 9 [file 41598_2025_20920_MOESM9_ESM.docx]

| **Parameter** | **Pre MI (pg/ml)** | **Post MI (pg/ml)** | **Post MI- endpoint (pg/ml)** |
| --- | --- | --- | --- |
| **Whole sample (n=24)** | | | |
| **cTn-I** | **50**  [47; 334] * | **11156**  [5926; 20739] | **47**  [38; 50] * |
| **TNF-ꭤ** | **1214**  [461; 1383] | **593**  [86; 1181] | **770**  [73;1596] |
| **IL6** | **691**  [497; 1126] | **510**  [321; 724] | **552**  [355; 840] |
| **IL1β** | **1453**  [921; 2772] | **827**  [484; 1342] | **985**  [639; 1451] |
| **Females (n=11)** | | | |
| **cTn-I** | ***101**  [47; 474] | **11918**  [8498; 12616] | ***50**  [38; 50] |
| **TNF-ꭤ** | **1206**  [247; 1387] | **621**  [78; 1192] | **751**  [377; 1655] |
| **IL6** | **526**  [412; 706] | **392**  [187; 690] | **620**  [333; 858] |
| **IL1β** | 1187  [901; 2004] | 709  [58; 937] | 990  [390; 2598] |
| **Males (n=13)** | | | |
| **cTn-I** | **47**  [47; 392] | **8957**  [5427; 25143] | **47**  [38; 47] |
| **TNF-ꭤ** | **1222**  [561; 1393] | **566**  [168; 1308] | **790**  [72; 1607] |
| **IL6** | **1052**  [498; 1292] | **553**  [460; 800] | **496**  [358; 840] |
| **IL1β** | **2151**  [1027; 3106] | **953**  [639; 1446] | **930**  [697; 1103] |

**Supplementary file S8. Values of troponin I (cTn-I), Tumor Necrosis Factor Alpha (TNF-ꭤ), Interleukin 6 (IL6) and Interleukin 1 Beta (IL1β) recorded at each day (Pre MI; Post MI; Post MI-endpoint) for each sex.** Results are reported as median and interquartile range [25^th^; 75^th^] * 1 missing values. Unit of measure: picograms/milliliters (pg/ml)
