# Supplementary material for: Unravelling pain in Göttingen Minipigs undergoing experimentally induced closed-chest myocardial infarction: a prospective cohort study
Source: Sci Rep. 2025 Oct 22;15:36934. doi: 10.1038/s41598-025-20920-y (PMC12546812; doi:10.1038/s41598-025-20920-y)

**Supplementary table S10. Values of heart rate (HR) and respiratory rate (RR) recorded at each day (Pre MI, Post MI and Post MI-endpoint) for the whole samples (n=24).**

| **Parameter** | **Day** | | |
| --- | --- | --- | --- |
|  | **Pre MI**  (n=24) | **Post MI**  (n=24) | **Post MI- endpoint** (n=24) |
| **HR (bpm)** | **90**  [87; 104] | **80**  [76; 88] | **84**  [80; 96] |
| **RR (bpm)** | **30**  [20; 40] | **22**  [20; 17] | **32**  [24; 36] |

Results are reported as median and interquartile range [25^th^; 75^th^]. Unit of measure: bpm: beats per minute (for heart rate) and breath per minute (for respiratory rate)

**Supplementary fig S10. Box plots showing values of heart rate (HR; unit of measure: beats per minute) for each day (Pre-MI, Post MI and Post MI- endpoint) in the whole sample (n=24) (A) and for each sex (f: females; m: males) (females= 11 minipigs, males= 13 minipigs).**

bpm: beats per minute * Statistically significant difference compared to Pre MI


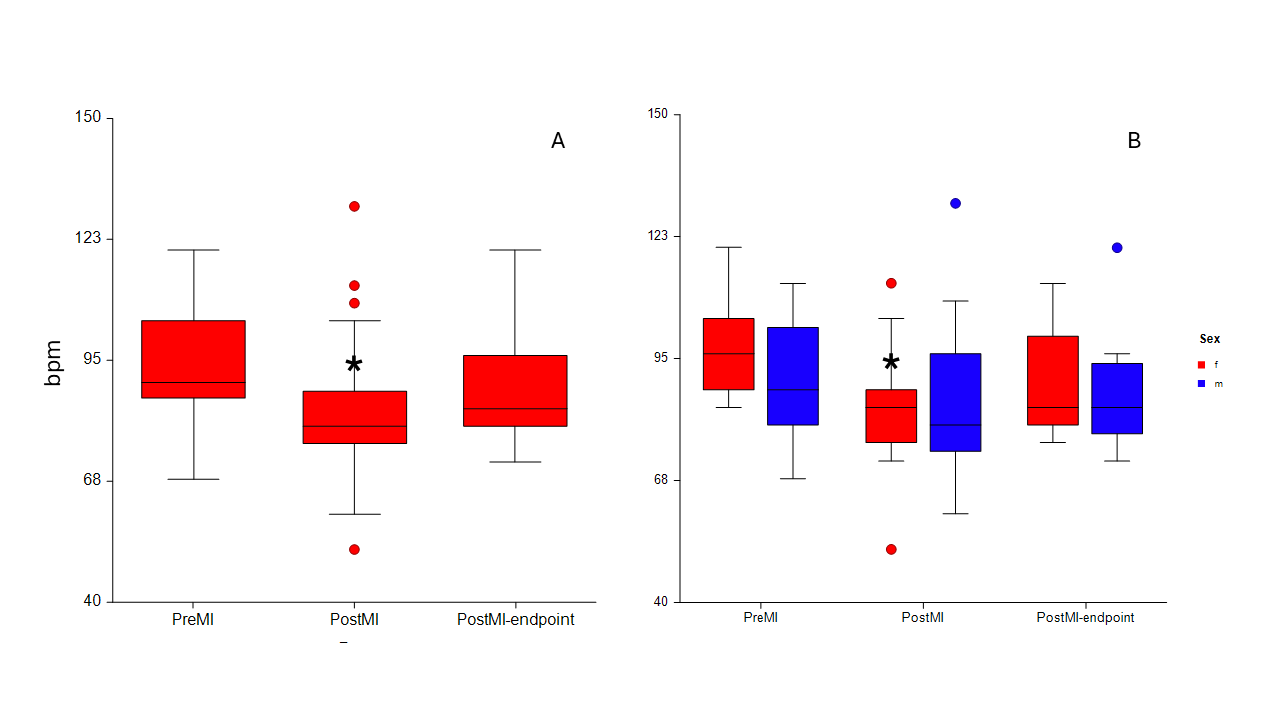

Supplement: Supplementary file 10 — Supplementary Material 10 [file 41598_2025_20920_MOESM10_ESM.docx]
