# Supplementary material for: Unravelling pain in Göttingen Minipigs undergoing experimentally induced closed-chest myocardial infarction: a prospective cohort study
Source: Sci Rep. 2025 Oct 22;15:36934. doi: 10.1038/s41598-025-20920-y (PMC12546812; doi:10.1038/s41598-025-20920-y)
Supplement: Supplementary file 11 — Supplementary Material 11 [file 41598_2025_20920_MOESM11_ESM.docx]

**Supplementary file S16:** Score used in the immediate post operative period (Post MI) following myocardial infarction induction (up to 12 hours after sternal recumbency)

**PHYSIOLOGICAL PARAMETERS**

HEART RATE (HR) AND RESPIRATORY RATE (RR) (0-2)

**Baseline** = **Parameters recorded pre-operatively**

1. HR and RR comparable to baseline (increase ≤ 20% from baseline)
2. Moderate increase of only one parameter (either HR or RR), between 20% and 30% of the normal range
3. Severe increase (more than 30%) of only one or both parameters.

TEMPERATURE (0-2)

1. Within the normal range (37-38°C± 0.3°C)
2. Moderate increase of temperature (38.3-39°C)
3. Severe increase of temperature (>39°C)

ARTERIAL BLOOD PRESSURE (0-2)

1. Systolic arterial pressure (SAP) within the normal range of variation (110- 160 mmHg)
2. Moderate increase of the SAP (161≥x≤200 mmHg)
3. Severe increase of the SAP (> 200 mmHg)

Measure IBP (invasive blood pressure) whenever applicable. If not possible, measure systolic pressure with Doppler technique.

SpO_2_

SpO2 will be evaluated, and hypoxemia will be treated with supplemental oxygen, but no analgesic score will be attributed to this parameter.

*Total score of physiological parameters: 6*

**BEHAVIORAL PARAMETERS**

APPEARANCE (0-1)

1. Normal appearance
2. Salivation

LYING AND RESTLESSNESS (0-3)

1. Normal lying
2. Lying guarding one part of the body/moves without external stimulation
3. Move often/ poor wake sleep times
4. Continuous pacing in the box

TREMORS/ SPASMS (0-2)

1. No evidence of tremors/spasms
2. Tremors/spasms in one part of the body
3. Generalized tremors/spasms.

VOCALISATION (0-1)

1. No vocalisation or vocalization related to food/interest in human approaching
2. Vocalisation without a purpose/unmotivated excitation

FOOD INTEREST (0-3)

1. Normal appetite
2. Reduced appetite, eat special food
3. Reduced appetite independently of the food
4. No appetite

AGITATION AT HUMAN APPROACHING (0-3)

1. Curious, interactive, may vocalize
2. Moves away when approached
3. Biting and aggressive when approached
4. Stay immobile and disinterested

RESPONSE TO TOUCH (0-2)

Referred pain will be checked on *neck, head, jam, thorax, forelimbs.*

1. No response
2. Mild response: the animal looks uncomfortable and worried; retraction of the interested body part.
3. Severe response: escape reaction

*Total score of behavioral parameters: 15*

**INTERVENTIONS**

**The animal will be continuously monitored by a veterinarian until achieving sternal recumbency and will be then regularly checked by the veterinarians or one of the designed members (deputy evaluators) of the ESF team at least until 12 hours post-surgery.**

**All minipigs will receive meloxicam 0.4 mg/kg IV at the end of surgery and for the following 3 days (SID) independently of the score sheet.**

TIME POINT “**EXTUBATION**” (Total score:11):

**If the score is ≤ 3/11 no intervention is needed.**

- With a score of 4-6: inject Morphine 0.2 mg/kg IM. Perform a blood gas analysis. Reevaluate the animal at the following time point.
- With a score 7-11: Inject Morphine 0.5 mg/kg IM.

TIME POINT “**STERNAL RECUMBENCY**” (Total score: 17)

**If the score is ≤ 3/17 no intervention is needed.**

- With a score of 4-10: administer rescue analgesia: Morphine 0.2 mg/kg IM.
- With a score of 11-17: administer rescue analgesia: Morphine 0.5 mg/kg IM.

*If morphine has been administered at this time point, re-evaluate the animal at time point “1 hour after sternal recumbency”.*

FROM THE “**15 MINUTES AFTER STERNAL RECUMBENCY**” TIME POINT TO THE “**12 HOURS AFTER STERNAL RECUMBENCY**” TIME POINT

No additional intervention needed if the score is **≤** **7/21**.

1. With a score of 8-11: inject Morphine 0.2 mg/kg IM. Repeat the score after 30 minutes. If the score is reduced of at least 2 points, wait further 30 minutes and re-evaluate. If no changes are present, proceed as B)
2. With a score of 12-16: inject Morphine 0.2 mg/kg IM and Flunixin 1 mg/kg. Repeat the score after 30 minutes. If the score is reduced of at least 2 points, wait further 30 minutes and reevaluate. If no changes are present, proceed as C).
3. With a score of 17-21: inject Morphine 0.2 mg/kg IM and Flunixin 1 mg/kg and start a Dexmedetomidine 4 mcg/kg/h constant rate infusion (CRI) IV. Repeat the score after 30 minutes.

If the score is reduced of at least 2 points, wait further 30 minutes and re-evaluate. If no changes are present, exclude confounding factors. Perform an arterial blood gas analysis, and/or thoracic radiology, ECG and cardiac ultrasonography. Establish therapy and reevaluate after 60 minutes. If the score is reduced of at least 2 points, continue with therapy, wait further 60 minutes and re-evaluate.

If no changes are present, proceed with euthanasia.

*****

**- If during monitoring, ECG evaluation is consistent with ventricular tachycardia, treat with antiarrhythmics.**

**- If during monitoring, ECG evaluation is consistent with ventricular fibrillation treat once with electric defibrillation. If unresponsive, proceed with euthanasia.**

**- If the arrhythmia is unresponsive to treatments within 12 hours, proceed with euthanasia.**

**This should be applied to each time points.**
