# Supplementary material for: Unravelling pain in Göttingen Minipigs undergoing experimentally induced closed-chest myocardial infarction: a prospective cohort study
Source: Sci Rep. 2025 Oct 22;15:36934. doi: 10.1038/s41598-025-20920-y (PMC12546812; doi:10.1038/s41598-025-20920-y)
Supplement: Supplementary file 12 — Supplementary Material 12 [file 41598_2025_20920_MOESM12_ESM.docx]

**Supplementary file S15. *Feasibility score for evaluation of animal cooperation during mechanical and thermal thresholds assessment.***

| **Feasibility score** | **Description** |
| --- | --- |
| *0 –*  *Excellent cooperation* | Clear reaction to stimuli in at least 90% of the measurements |
| *1 –*  *Good cooperation* | Clear reaction to stimuli in at least 75% of the measurements |
| *2 –*  *Moderate cooperation* | Clear reaction to stimuli in at least 50% of the measurements |
| *3 –*  *Low cooperation* | Clear reaction to stimuli in less than 50% of the measurements |
