# Supplementary material for: Unravelling pain in Göttingen Minipigs undergoing experimentally induced closed-chest myocardial infarction: a prospective cohort study
Source: Sci Rep. 2025 Oct 22;15:36934. doi: 10.1038/s41598-025-20920-y (PMC12546812; doi:10.1038/s41598-025-20920-y)
Supplement: Supplementary file 13 — Supplementary Material 13 [file 41598_2025_20920_MOESM13_ESM.docx]

**Supplementary figure S7.** Feasibility score (FS) (0,1,2) recorded during mechanical thresholds (MT) assessment at each day (0= Pre MI; day 1= Post MI; day 45= Post MI- endpoint) for the whole sample (n=24).

**Supplementary figure S8.** Feasibility score (FS) (0,1,2) recorded during mechanical thresholds (MT) assessment at each day (0= Pre MI; day 1= Post MI; day 45= Post MI- endpoint) in female minipigs (n=11).


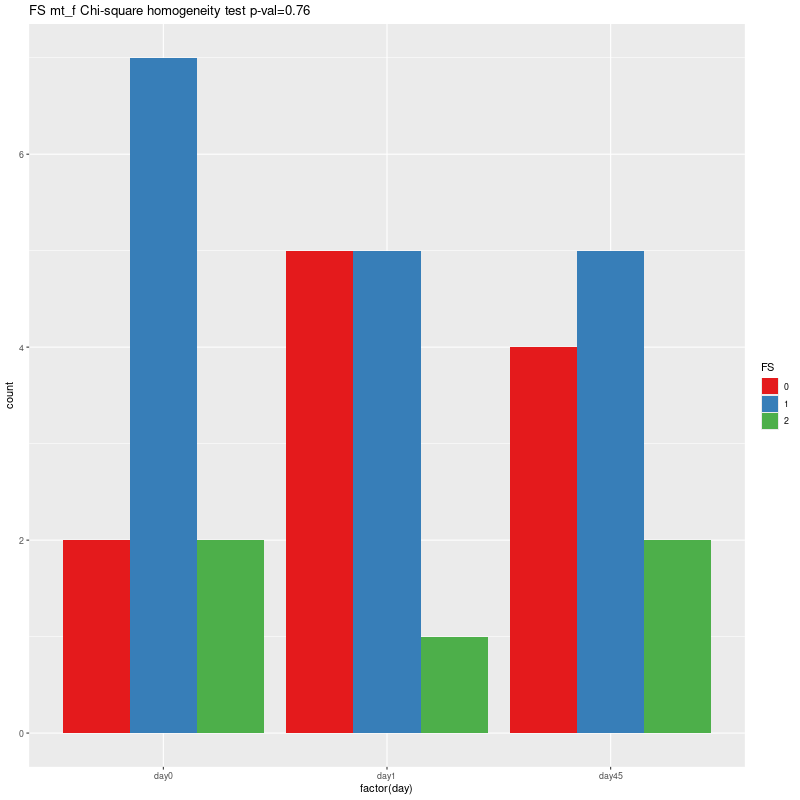


**Supplementary figure S9.** Feasibility score (FS) (0,1,2) recorded during mechanical thresholds (MT) assessment at each day (0= Pre MI; day 1= Post MI; day 45= Post MI- endpoint) in male minipigs (n=13).

**Supplementary figure S10.** Feasibility score (FS) (0,1,2) recorded during thermal thresholds (TT) assessment at each day (0= Pre MI; day 1= Post MI; day 45= Post MI- endpoint) in the whole sample (n= 24).

**Supplementary figure S11.** Feasibility score (FS) (0,1,2) recorded during thermal thresholds (TT) assessment in the different days (0= Pre MI; day 1= Post MI; day 45= Post MI- endpoint) in female minipigs (n=11).

**Supplementary figure S12.** Feasibility score (FS) (0,1,2) recorded during thermal thresholds (TT) assessment in the different days (0= Pre MI; day 1= Post MI; day 45= Post MI- endpoint) in male minipigs (n=13).
