# Supplementary material for: Unravelling pain in Göttingen Minipigs undergoing experimentally induced closed-chest myocardial infarction: a prospective cohort study
Source: Sci Rep. 2025 Oct 22;15:36934. doi: 10.1038/s41598-025-20920-y (PMC12546812; doi:10.1038/s41598-025-20920-y)
Supplement: Supplementary file 14 — Supplementary Material 14 [file 41598_2025_20920_MOESM14_ESM.docx]

**Supplementary file S14**: Score used from post operative day 1 to the study endpoint (Post MI-endpoint, 42± 3 after myocardial infarction induction)

**PHYSIOLOGICAL PARAMETERS**

Heart Rate (HR) and Respiratory Rate (RR) (0-2):

**Baseline** = **Parameters recorded pre-operatively.**

1. HR and RR comparable to baseline (increase ≤ 20% from baseline)
2. Moderate increase of only one parameter (either HR or RR), between 20% and 30% of the normal range
3. Severe increase (more than 30%) of only one or both parameters.

Temperature (0-2):

1. Within the normal range (37-38°C± 0.3°C)
2. Moderate increase of temperature (38.3-39°C)
3. Severe increase of temperature (> 39°C)

*Total score for physiological parameters: 4*

**SOLITARY PERFORMANCES**

Appearance: (0-1)

1. Normal appearance
2. Salivation

Lying and restlessness (0-3):

1. Normal lying
2. Lying guarding one part of the body/moves without external stimulation
3. Move often/poor wake sleep times
4. Continuous pacing around the pen/in the box

Food interest (0-3)

1. Normal appetite
2. Reduced appetite, eat special food
3. Reduced appetite independently of the food
4. No appetite

*Total score for solitary performances: 7*

**SOCIAL PERFORMANCES**

Aggression with co-mates (0-3):

1. Friendly
2. Moves away
3. Biting and aggressive when approached by other pigs
4. No aggression because immobility

Isolation (co-mates) (0-1):

1. Look actively for playing
2. Not interested in playing

Agitation at human approaching (0-3):

1. Curious, interactive, may vocalize
2. Moves away when approached
3. Biting and aggressive when approached
4. Stay immobile and disinterested

Desynchronisation (0-2):

1. Synchronised with the co-mates (same activity of the co-mates)
2. Desynchronised (different activity from that of most co-mates)

Response to touch (0-2):

(Referred pain will be checked on neck, head, jam, thorax, forelimbs

1. No response
2. Mild response: the animal looks uncomfortable and worried; retraction of the interested body part.
3. Severe response: escape reaction

*Total score for social performances: 11*

**INTERVENTIONS**

**The animals will be evaluated once per day until POD 7 and 3 times a week for the entire duration of the experiment.**

**All the animals will be treated with Metacam 0.4 mg/kg SID per OS and Omeprazole 1 mg/kg BID per OS until POD3.**

No additional intervention needed if the score is **≤ 4/22**.

1. With a score of 5-8: administer Flunixin 1 mg/kg IM.

- If the score is not diminished after 2 hours, exclude the presence of secondary diseases (complete the “score sheet for secondary disease- SDSS”). If SSSD is negative, inject Morphine 0.2 mg/kg IM.

Repeat the LTSS after two hours.

- If no changes, proceed with hematological examination (complete blood count (CBC) and serum biochemistry) and diagnostic imaging.

1. With a score of 9-15: complete the score sheet SDSS and if it is negative inject Morphine 0.2 mg/kg IM and Flunixin 1.5 mg/kg IM.

Repeat both scores (LTSS and SDSS) after 2 hours.

- If after analgesia the LTSS is reduced to 5-8, place Transdermal Buprenorphine Patch (*) 30 mcg/h and Flunixin 1 mg/kg IM.

- If there are no changes, perform the SSSD and if negative the animal will be promptly brought to the Experimental surgery facility (ESF) intensive care unit (ICU) where oxygen therapy (flow by or face mask) hematological examination (complete blood count -CBC- and serum biochemistry) and diagnostic imaging will be performed as well as further pain treatment (Dexmedetomidine and Fentanyl CRI).

- If heart insufficiency, kidney insufficiency, hepatic insufficiency is confirmed, proceed with immediate euthanasia

- If excluded, re-evaluate the minipig after 1 hour of analgesic CRI. If no changes, proceed with euthanasia.

- If after analgesia the LTSS is reduced to 5-8, place Transdermal Buprenorphine Patch 30 mcg/h, inject Flunixin 1 mg/kg IM and re-integrate into the herd.

C) With a score ≥16: perform the SSSD:

1. **Evident pain source** (e.g. contusion, wound…) and the SSSD is negative:

- Inject Morphine 0.2 mg/kg IM and bring promptly the animal to the ESF-ICU.

If after 1 hour from the administration of analgesia the LTSS is reduced to 9-15, continue with Dexmedetomidine or Fentanyl CRI and re-evaluate the animal every hour up to 12 hours.

If the score is reduced to 5-8, place Transdermal Buprenorphine Patch 30 mcg/h and inject Flunixin 1 mg/kg IM and re-integrate the animal into the herd.

1. **No evident pain source**:

- SSSD positive for heart insufficiency: treat with and Furosemide 2 mg/kg IM and then bring promptly the animal to ESF-ICU, where oxygen therapy (flow-by or face mask), hematological examination (CBC and serum biochemistry) and diagnostic imaging will be performed.

If heart insufficiency is confirmed, the animal will be treated with a second bolus Furosemide, 2 mg/kg IV. If after 1 hour from the administration of the second bolus, the score is not <16, proceed with euthanasia.

If after 1 hour, the score is 9-15, start Furosemide constant rate infusion (CRI) 1 mg/kg/h for 6 hours.

Repeat the LTSS each hour.

- If after 6 hours LTSS is not <9, proceed with euthanasia.
- If after 6 hours LTSS is <9, stop Furosemide CRI and re-evaluate after 6 h. If the LTSS is still <9, re-integrate into the herd.
- SSSD positive for infection: Treat with antibiotics therapy for 7 days and place Transdermal Buprenorphine Patch 30 mcg/h.
- SSSD negative: kidney insufficiency or hepatic insufficiency need to be evaluated by hematological examination.

If confirmed, proceed with immediate euthanasia.

**Weight**

The body weight calculation should be performed daily up to POD 3 and then once a week up to day 42. If there is the evidence of a reduction of 10% from the baseline (day 0) consider if the amount of food is sufficient and perform hematological examination.

(*) Place the Transdermal Buprenorphine Patch on the dorsal area approximately 1-2 cm from the midline (right and/or left) between the 12^th^ thoracic and 2^nd^ lumbar vertebrae.

- Before patch application, the skin should be shaved and cleaned with saline.

- After patch application, the patch should be covered with an occlusive and flexible bandage.

**The decision tree is used only by the veterinarian in charge.**

**SSSD POSITIVE FOR HEART INSUFFICIENCY**

- **Whenever the score sheet for secondary disease is positive for heart insufficiency, the animal will receive Furosemide 2 mg/kg IV and, subsequently will be promptly brought to ESF-ICU under veterinary supervision.**

**Once the animal is in ESF-ICU, if there will be the evidence of cardiac insufficiency (based on clinical and instrumental examination), it will be treated with a second bolus of Furosemide 2 mg/kg IV (if it was unresponsive to the first bolus) and re-evaluate after 1 hour.**

**If the minipig will be considered again unresponsive to treatment, euthanasia will be performed.**

**SSSD POSITIVE FOR INFECTION**

- **In case of the SSSD is indicative of an infection, a combination antibiotic (e.g. amoxicillin/clavulanic acid, 20 mg/Kg per OS or IM/IV, up to 4x/day) will be administered for 7 days at the farm.**

| Date: | Animal number: | SSSD positive for: | Evaluator: |
| --- | --- | --- | --- |
|  |  | Heart insufficiency  Infection |  |
|  |  | Infection  Heart insufficiency |  |
|  |  | Heart insufficiency  Infection |  |
|  |  | Infection  Heart insufficiency |  |
