# Supplementary material for: Unravelling pain in Göttingen Minipigs undergoing experimentally induced closed-chest myocardial infarction: a prospective cohort study
Source: Sci Rep. 2025 Oct 22;15:36934. doi: 10.1038/s41598-025-20920-y (PMC12546812; doi:10.1038/s41598-025-20920-y)
Supplement: Supplementary file 15 — Supplementary Material 15 [file 41598_2025_20920_MOESM15_ESM.docx]

**Supplementary file S13:** Score used during acclimatization period and before myocardial infarction induction.

Heart Rate (HR) and Respiratory Rate (RR) (0-2):

HR and RR comparable to baseline (increase ≤ 20% from baseline)

1. Moderate increase of only one parameter (either HR or RR), between 20% and 30% of the normal range
2. Severe increase (more than 30%) of only one or both parameters.

Baseline = Parameters taken after the arrival from the facility of origin

Temperature (0-2):

1. Within the normal range (37-38°C± 0.3°C)
2. Moderate increase of temperature (38.3-39°C)
3. Severe increase of temperature (>39°C)

Diarrhea (0-1):

1. Absent
2. Present

Ocular/ Nasal discharge (0-1):

1. Absent
2. Present

Cough (0-1):

1. Absent
2. Present

*Total score of physiological parameters: 7*

**SOLITARY PERFORMANCES**

Appearance (0-1)

1. Normal appearance
2. Salivation

Lying and restlessness (0-3)

1. Normal lying
2. Lying guarding one part of the body/moves without external stimulation
3. Move often/poor wake sleep times
4. Continuous pacing around the pen/in the box

Food interest (0-3)

1. Normal appetite
2. Reduced appetite, eat special food
3. Reduced appetite independently of the food
4. No appetite

*Total score of solitary performances: 7*

**SOCIAL PERFORMANCES**

Aggression with co-mates (0-3)

1. Friendly
2. Moves away
3. Biting and aggressive when approached by other pigs
4. No aggression because immobility

Isolation (co-mates) (0-1)

1. Look actively for playing
2. Not interested in playing

Desynchronisation (0-2)

1. Synchronised with the co-mates (same activity of the co-mates)
2. Desynchronised (different activity from that of most co-mates)

*Total score of social performances: 6*

**INTERVENTIONS**

**All the minipigs will be evaluated on arrival at farm and then three times per week, up to the end of acclimatisation period.**

If the score is ≤ 8/20, no intervention is needed.

- If presence of ***cough and ocular/nasal discharge***: suspect respiratory infection and a combination antibiotic (e.g. amoxicillin/clavulanic acid, 20 mg/Kg per OS or IM/IV, up to 4x/day) will be administered for 7 days at the farm.
- If presence of ***diarrhea***: perform fecal examination to detect parasites infestation. Then, treat as appropriate.

If without sign of cough, diarrhea and nasal/ocular discharge the score is more than 9:

- Control food quality and amount, animal’s management and perform hematological examination (complete blood count and serum biochemistry).
- Re-evaluate after 1 day.
