# Supplementary material for: Unravelling pain in Göttingen Minipigs undergoing experimentally induced closed-chest myocardial infarction: a prospective cohort study
Source: Sci Rep. 2025 Oct 22;15:36934. doi: 10.1038/s41598-025-20920-y (PMC12546812; doi:10.1038/s41598-025-20920-y)

**Supplementary figure S1.** Box plots showing modifications of mechanical thresholds (range 0-101 Newton) at each day, for each minipig (pink= female minipigs, blue = male minipigs). Legend: day 0: Pre MI, day 1: Post MI; day 45= Post MI- endpoint.

Pigs 6, 8, 16 and 17 received rescue analgesia at day 1 (Post MI).


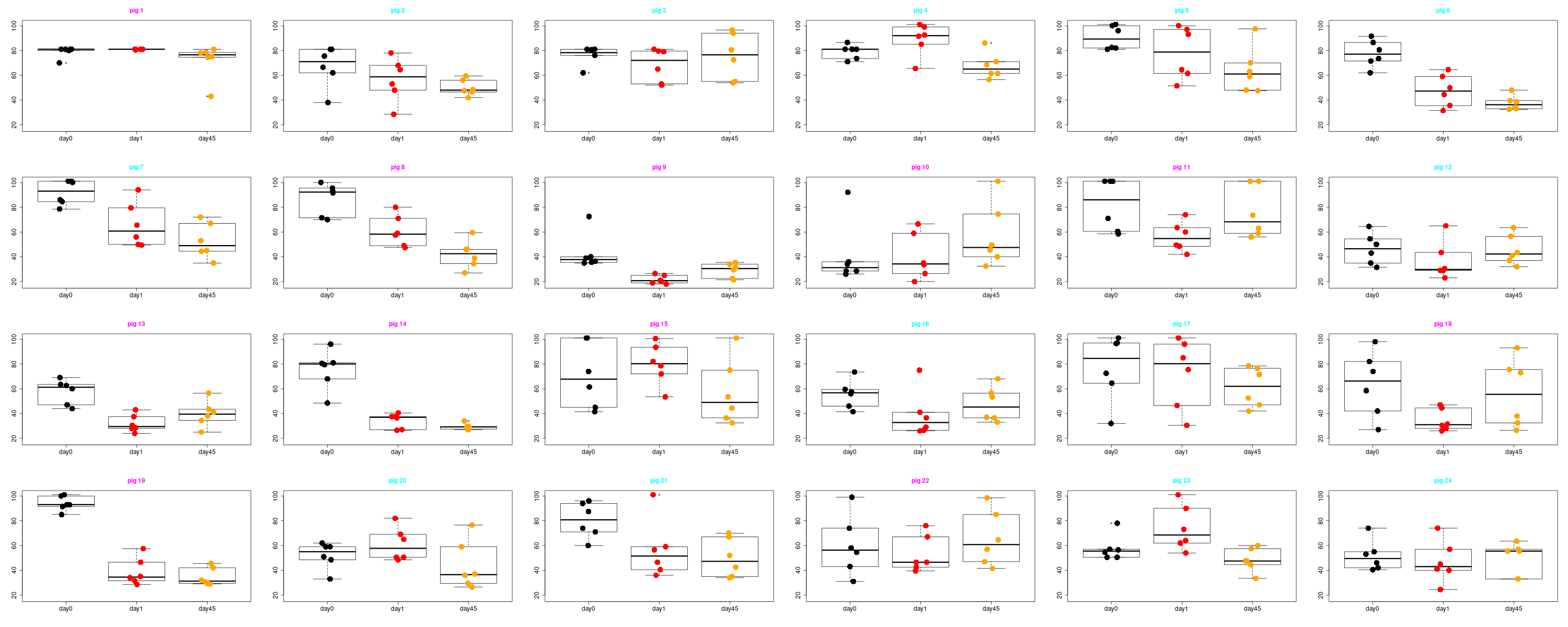


**Supplementary figure S2.** Box plots showing modifications of mechanical thresholds (range 0-101 Newton) at each day in female minipigs. Legend: day 0: Pre MI, day 1: Post MI; day 45= Post MI- endpoint. Pig 8 received rescue analgesia at day 1 (Post MI).


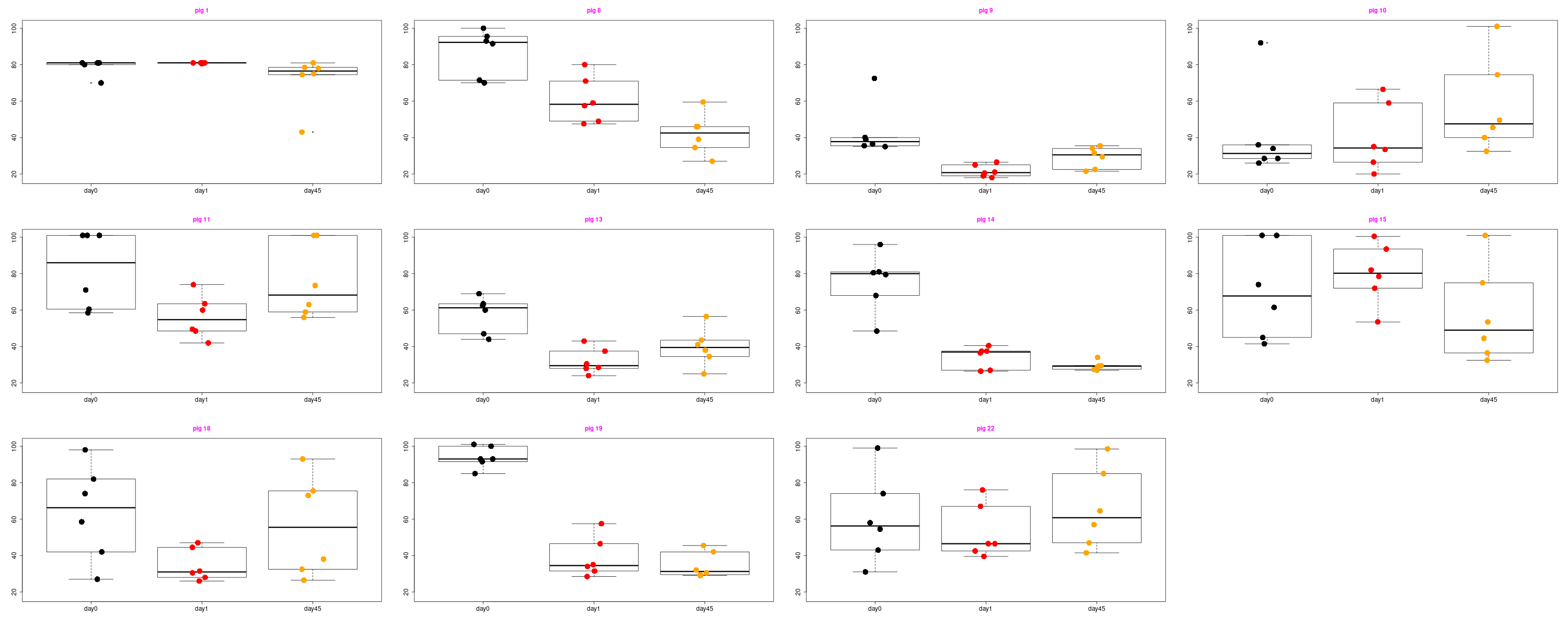


**Supplementary figure S3.** Box plots showing modifications of mechanical thresholds (range 0-101 Newton) at each day in male minipigs. Legend: day 0= Pre MI, day 1= Post MI, day 45= Post MI- endpoint. Pigs 6, 16, 17 received rescue analgesia at day 1 (Post MI).


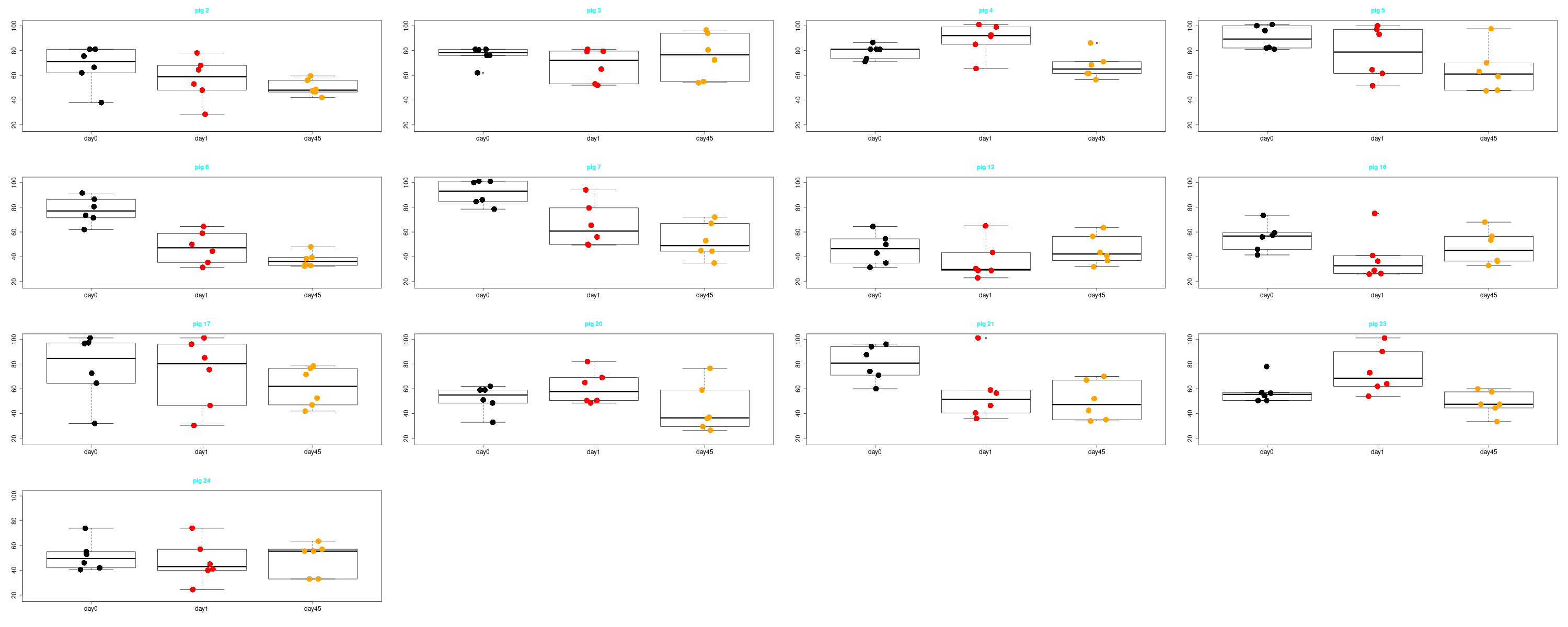


**Supplementary figure S4.** Box plots showing modifications of thermal thresholds (range 20-56 degrees Celsius) at each day for each minipig (pink= female minipig, blue = male minipig). Legend: day 0= Pre MI, day 1= Post MI, day 45= Post MI- endpoint.

Pigs 6, 8, 16 and 17 received rescue analgesia at day 1 (Post MI).


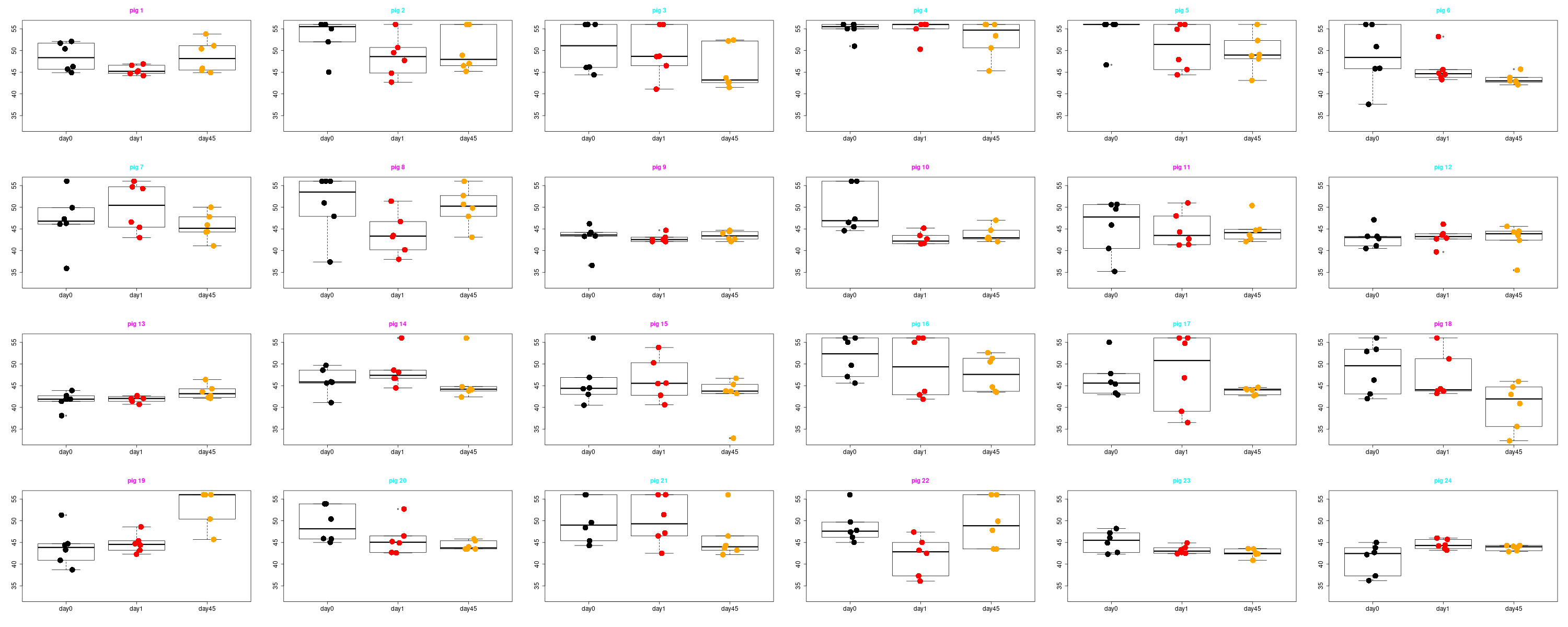


**Supplementary figure S5.** Box plots showing modifications of thermal thresholds (range 20-56 degrees Celsius) at each day in female minipigs. Legend: day 0= Pre MI, day 1= Post MI, day 45= Post MI- endpoint. Pig 8 received rescue analgesia at day 1 (Post MI).


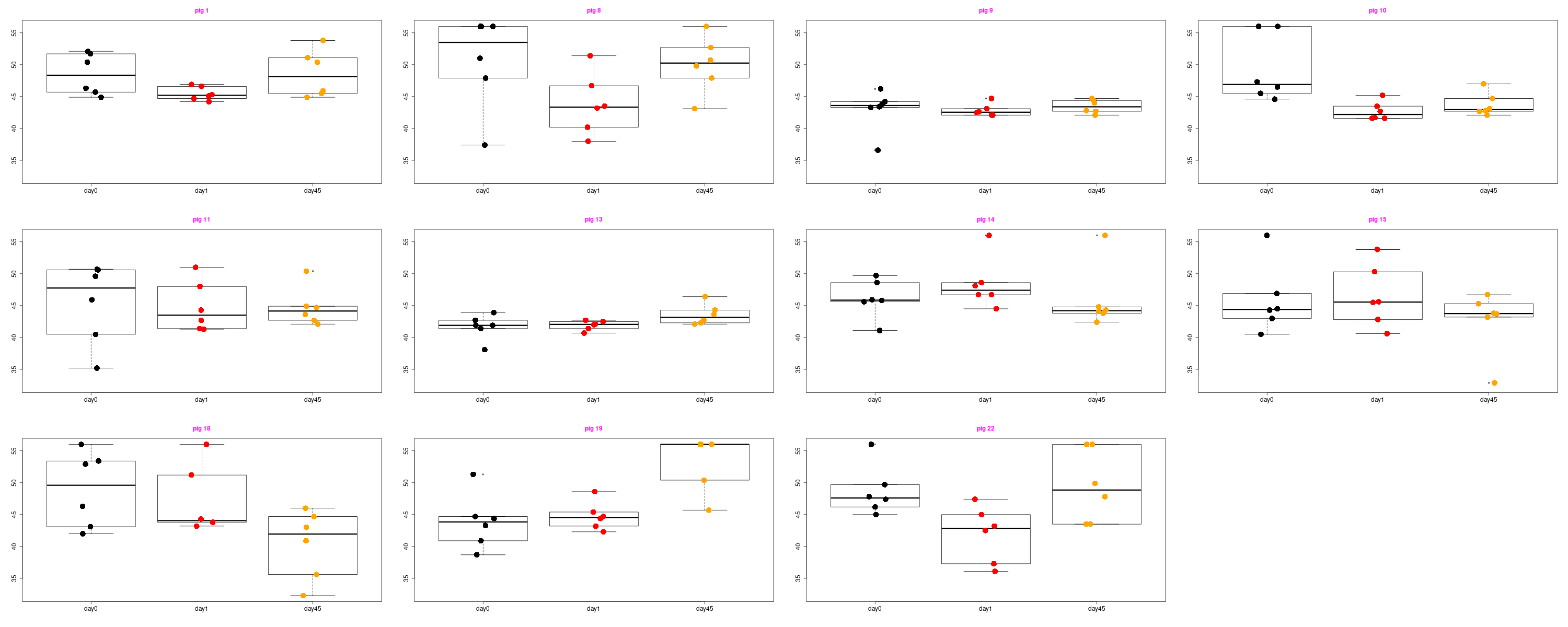


**Supplementary figure S6.** Box plots showing modifications of thermal thresholds (range 20-56 degrees Celsius) at each day in male minipigs. Legend: day 0= Pre MI, day 1= Post MI, day 45= Post MI- endpoint. Pigs 6, 16, 17 received rescue analgesia at day 1 (Post MI).


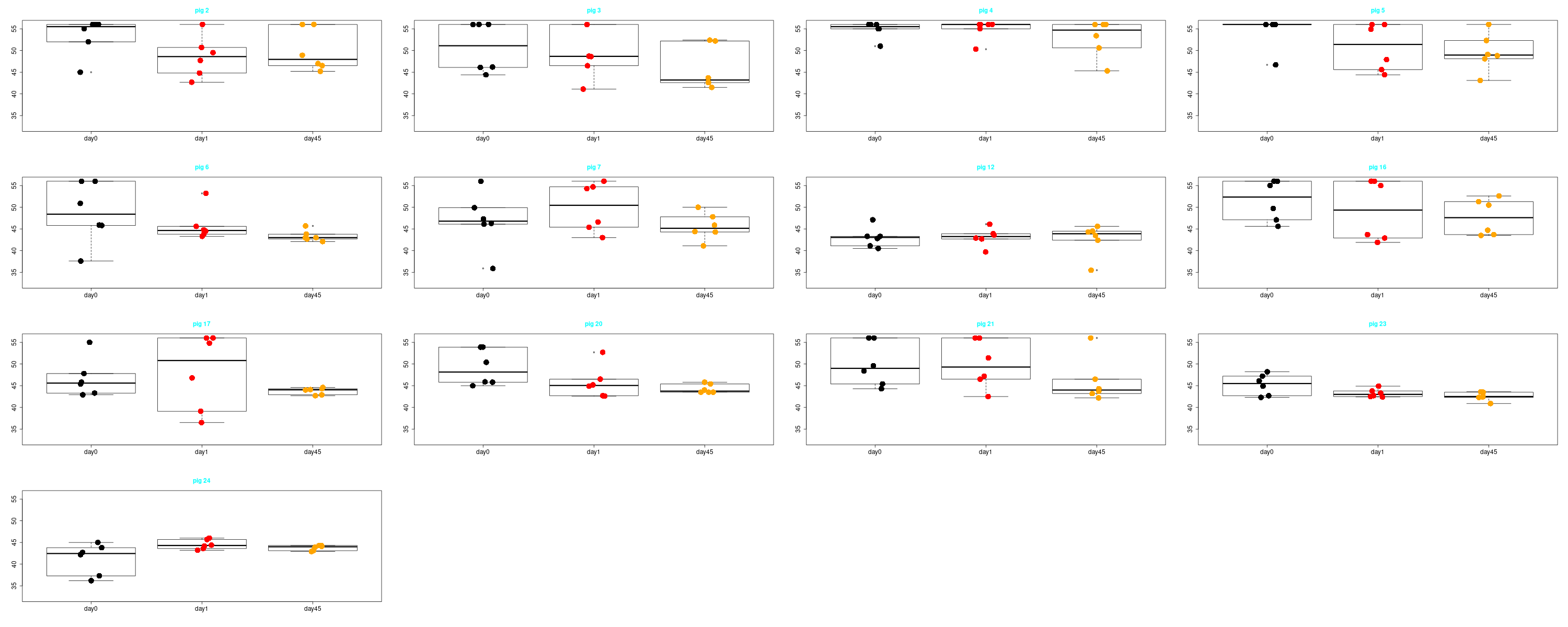

Supplement: Supplementary file 16 — Supplementary Material 16 [file 41598_2025_20920_MOESM16_ESM.docx]
